# CD8^+^ T-Cells Expressing Interferon Gamma or Perforin Play Antagonistic Roles in Heart Injury in Experimental *Trypanosoma Cruzi*-Elicited Cardiomyopathy

**DOI:** 10.1371/journal.ppat.1002645

**Published:** 2012-04-19

**Authors:** Jaline Coutinho Silverio, Isabela Resende Pereira, Márcio da Costa Cipitelli, Nathália Ferreira Vinagre, Maurício Martins Rodrigues, Ricardo Tostes Gazzinelli, Joseli Lannes-Vieira

**Affiliations:** 1 Laboratório de Biologia das Interações, Instituto Oswaldo Cruz, Fiocruz, Rio de Janeiro, Brazil; 2 Departamento de Microbiologia, Imunologia e Parasitologia, UNIFESP, São Paulo, Brazil; 3 Laboratório de Imunoparasitologia, Instituto Rene Rachou, Fiocruz, Minas Gerais, Brazil; 4 Departamento de Imunologia e Bioquímica, ICB, UFMG, Minas Gerais, Brazil; National Institutes of Health, United States of America

## Abstract

In Chagas disease, CD8^+^ T-cells are critical for the control of *Trypanosoma cruzi* during acute infection. Conversely, CD8^+^ T-cell accumulation in the myocardium during chronic infection may cause tissue injury leading to chronic chagasic cardiomyopathy (CCC). Here we explored the role of CD8^+^ T-cells in *T. cruzi*-elicited heart injury in C57BL/6 mice infected with the Colombian strain. Cardiomyocyte lesion evaluated by creatine kinase-MB isoenzyme activity levels in the serum and electrical abnormalities revealed by electrocardiogram were not associated with the intensity of heart parasitism and myocarditis in the chronic infection. Further, there was no association between heart injury and systemic anti-*T. cruzi* CD8^+^ T-cell capacity to produce interferon-gamma (IFNγ) and to perform specific cytotoxicity. Heart injury, however, paralleled accumulation of anti-*T. cruzi* cells in the cardiac tissue. In *T. cruzi* infection, most of the CD8^+^ T-cells segregated into IFNγ^+^ perforin (Pfn)^neg^ or IFNγ^neg^Pfn^+^ cell populations. Colonization of the cardiac tissue by anti-*T. cruzi* CD8^+^Pfn^+^ cells paralleled the worsening of CCC. The adoptive cell transfer to *T. cruzi*-infected *cd8* ^−/−^ recipients showed that the CD8^+^ cells from infected *ifn*γ^−/−^ *pfn* ^+/+^ donors migrate towards the cardiac tissue to a greater extent and caused a more severe cardiomyocyte lesion than CD8^+^ cells from *ifnγ* ^+/+^ *pfn* ^−/−^ donors. Moreover, the reconstitution of naïve *cd8* ^−/−^ mice with CD8^+^ cells from naïve *ifnγ* ^+/+^ *pfn* ^−/−^ donors ameliorated *T. cruzi*-elicited heart injury paralleled IFNγ^+^ cells accumulation, whereas reconstitution with CD8^+^ cells from naïve *ifnγ* ^−/−^ *pfn* ^+/+^ donors led to an aggravation of the cardiomyocyte lesion, which was associated with the accumulation of Pfn^+^ cells in the cardiac tissue. Our data support a possible antagonist effect of CD8^+^Pfn^+^ and CD8^+^IFNγ^+^ cells during CCC. CD8^+^IFNγ^+^ cells may exert a beneficial role, whereas CD8^+^Pfn^+^ may play a detrimental role in *T. cruzi*-elicited heart injury.

## Author Summary

Chagas disease, a neglected tropical disease that is caused by *Trypanosoma cruzi*, afflicts between 8 and 15 million people in Latin America. Anti-parasite immunity allows for acute phase survival; however, approximately 30% of patients present chronic chagasic cardiomyopathy (CCC) with parasite persistence and CD8-enriched myocarditis at 10 to 30 years post-infection. The comprehension of the pathogenesis of Chagas' heart disease may open a new avenue of therapy for CCC. Here, we explored the role of CD8^+^ T-cells in heart injury in C57BL/6 mice that were infected with the Colombian strain of *T. cruzi*. In infected mice, most of the CD8^+^ T-cells were segregated into CD8^+^ interferon-gamma (IFNγ)^+^perforin (Pfn)^neg^ and CD8^+^IFNγ^neg^Pfn^+^ cell populations. Importantly, the enrichment of the chronic myocarditis in anti-parasite CD8^+^Pfn^+^ cells paralleled the worsening of CCC. CD8^+^ cells from infected *ifnγ* ^−/−^ *pfn* ^+/+^ donors migrated towards the cardiac tissue to a greater extent than did CD8^+^ cells from *ifnγ* ^+/+^ *pfn* ^−/−^ donors. Moreover, accumulation of IFNγ^+^ cells in the cardiac tissue ameliorated cardiomyocyte lesion, whereas enrichment in CD8^+^Pfn^+^ cells aggravated cardiomyocyte injury. Therefore, our data suggest that CD8^+^IFNγ^+^ cells are beneficial, whereas CD8^+^Pfn^+^ cells are detrimental in *T. cruzi*-elicited heart injury.

## Introduction

*Trypanosoma cruzi* is the intracellular protozoan that causes American trypanosomiasis, which is also known as Chagas disease. In Latin America, 8–15 million people are estimated to currently be infected with this organism [1]. While 50% of *T. cruzi*-infected individuals suffer from an indeterminate form of the disease, the other 50% develop the digestive or the mild-to-severe cardiac form of Chagas disease between 10 and 30 years post-infection [2]. There is an emerging consensus that the pathology of chronic chagasic cardiomyopathy (CCC) is associated with parasite persistence and an imbalanced host immune response that favors chronic heart inflammation [3]. However, the cellular mechanisms leading to tissue damage in CCC are unknown.

CD8^+^ T-cells are crucial for *T. cruzi* dissemination control during the acute infection phase [4]. Based on the predominance of CD8^+^ T-cells in the cardiac inflammatory infiltrates of CCC patients [5] and chronically infected mice [6], the participation of a portion of these heart-invading cells in the immunopathology has been proposed [7]. CCC is absent or is less severe in patients with a significantly higher frequency of circulating interferon-gamma (IFNγ)-producing CD8^+^ T-cells that are specific for *T. cruzi* [8], [9]. In addition, there is a correlation between the number of IFNγ-producing cells and the lack of *T. cruzi* antigens in the heart lesions of CCC patients [10]. Adopting experimental murine models, we confirmed the presence of IFNγ mRNA and protein [6], [11], [12] in the cardiac tissue at the different stages of *T. cruzi* infection. However, there is no clear association between CD8-enriched myocarditis, which occurs in an IFNγ-containing milieu [12], and heart injury. Conversely, infiltrating CD8^+^ cells expressing granzyme A but devoid of the natural killer cell marker CD57 were in contact with myocardial cells in heart biopsies from CCC patients [13], suggesting a role for cytolytic CD8^+^ T-cells (CTL) in cardiomyocyte lesion. Corroborating these data, the deficiency of perforin (Pfn), a component of the CTL machinery [14], resulted in less severe cardiomyocyte lesion and electrical abnormalities during chronic *T. cruzi* infection [7].

CD8^+^ T-cells mediate protection against infection through the secretion of cytokines, such as IFNγ and tumor necrosis factor (TNF), and through CTL activity via the release of cytotoxic granules containing granzymes, granulysins and Pfn [14]. In humans, CD8^+^ T-cells are functionally segregated into inflammatory (IFNγ^+^Pfn^neg^) and cytotoxic (IFNγ^neg^Pfn^+^) effectors, which may influence the outcome of an infectious process [15]. Therefore, we investigated whether CD8^+^ T-cell effector activities are segregated into distinct CD8^+^ populations of inflammatory (IFNγ^+^) and cytotoxic cells (Pfn^+^) during a *T. cruzi* infection. Furthermore, it is reasonable to propose that the functional segregation of CD8^+^ T-cells *in vivo* has distinct implications for parasite control and the immunopathology of chronic cardiomyopathy. Therefore, adopting a murine model of chronic *T. cruzi*-elicited CD8-enriched myocarditis, we analyzed CD8^+^ T-cells to clarify whether they are multifunctional (IFNγ^+^Pfn^+^) or segregated into inflammatory (IFNγ^+^) and cytotoxic (Pfn^+^) cells. In addition, we examined the ability of CD8^+^ T-cells that selectively express IFNγ or Pfn to colonize the cardiac tissue and investigated whether they play a role in parasitism control or cardiac tissue injury during *T. cruzi* infection.

## Results

### Evolution of *T. cruzi*-elicited cardiomyocyte lesion and electrical abnormalities were not associated with the intensities of parasitism and CD8-enriched myocarditis

**Figure 1:**
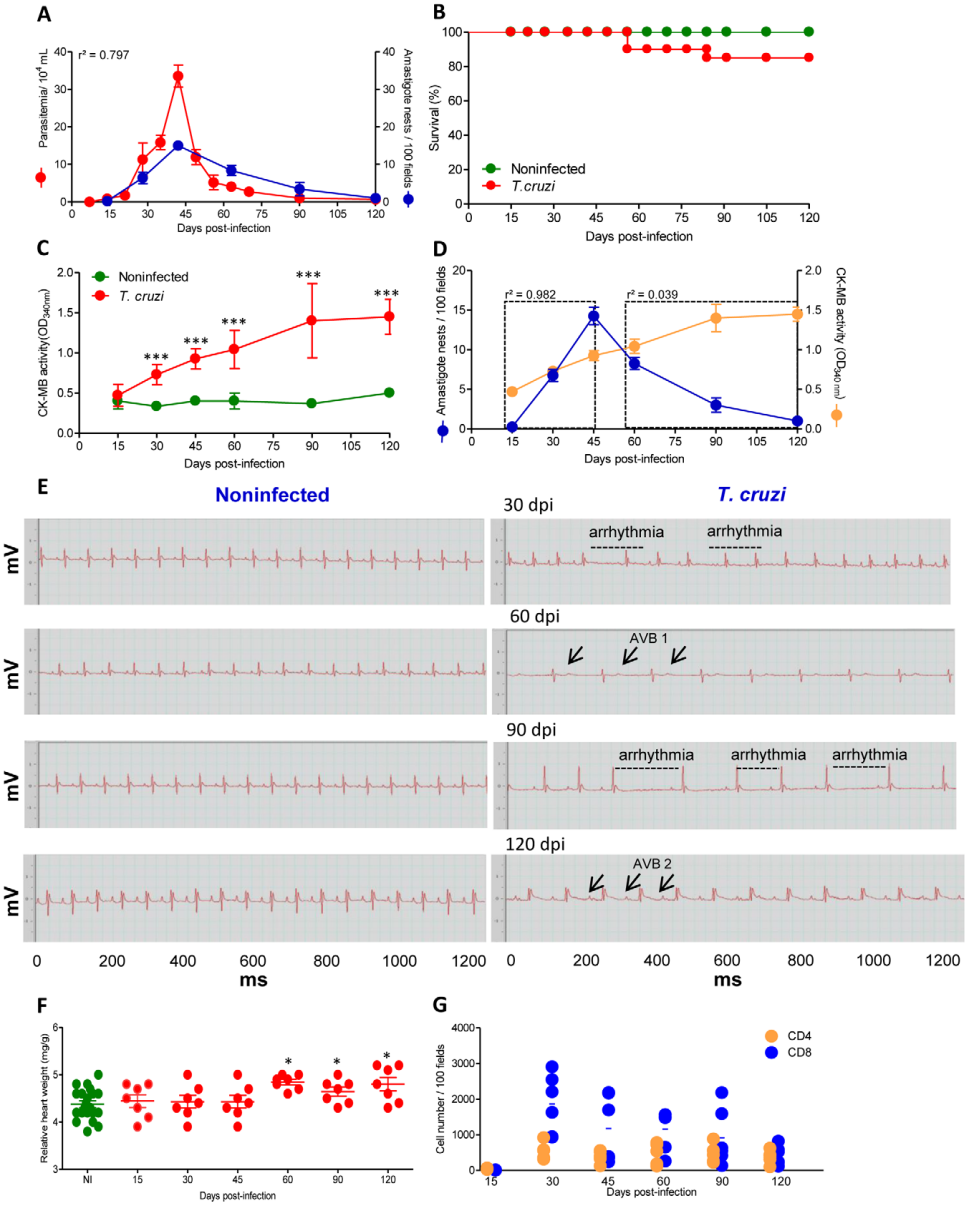
C57BL/6 mice infected with the Colombian strain of *T. cruzi* develop chronic cardiomyopathy. Mice were infected with 100 bt of the Colombian strain of *T. cruzi* and parasitological and clinical parameters were evaluated. (**A**) Kinetics of parasitemia and cardiac parasitism. (**B**) Survival rate. (**C**) CK-MB activity levels in the serum of noninfected and *T. cruzi*-infected mice. (**D**) Positive correlation between cardiac tissue parasitism and CK-MB activity levels during the acute phase (r^2^ = 0.982) but not during the chronic phase (r^2^ = 0.039) of infection. (**E**) Representative ECG register segments of 1200 ms of sex- and age-matched noninfected (NI) controls and *T. cruzi*-infected C57BL/6 mice at 30, 45, 60, 90 and 120 dpi, showing arrhythmia and first and second degree atrioventricular block (AVB1, AVB2; arrows) in infected mice. (**F**) Relative heart weight (mg/g) of noninfected (NI, pool of three age-matched controls per analyzed point) and *T. cruzi*-infected C57BL/6 mice at 15, 30, 45, 60, 90 and 120 dpi. (**G**) Numbers of CD4^+^ and CD8^+^ cells in the cardiac tissue during acute and chronic *T. cruzi* infection were counted after immunohistochemistry staining. Each circle represents an individual mouse. These data represent three independent experiments. ^*^, *p*<0.05, ^**^, *p*<0.01, and ^***^, *p*<0.001 comparing NI controls and *T. cruzi*-infected mice.

Initially, we investigated whether there was an association between the evolution of heart injury and parasite load in *T. cruzi*-infected C57BL/6 mice. The first peripheral blood circulating parasites were detected at 14 dpi, marking the onset of the acute infection phase. The peak of parasitemia was observed between 42 and 45 dpi and trypomastigotes were rarely found in the blood at 90 dpi, characterizing the onset of the chronic phase of infection (**Figure 1A**). With respect to cardiac parasitism, the first amastigote forms were detected at 15 dpi, and were frequently observed inside of myocytes from 30 dpi. The peak of heart parasitism coincided with the peak of parasitemia (42 dpi). After 60 dpi, the heart parasitism decreased and the parasite pseudocysts were barely detectable at 90 and 120 dpi (**Figure 1A**), although small pseudocysts and a few spots of parasite antigens were detected by IHS in every heart tissue section of all infected mice at every time point evaluated (data not shown). Therefore, in this model of *T. cruzi* infection, there was a correlation between parasitemia and heart parasitism during the acute and chronic phases of infection (r^2^ = 0.797, *p*<0.001). Approximately 80% of the infected mice survived the acute infection and developed the chronic infection (**Figure 1B**). To explore the heart injury, we evaluated creatine kinase cardiac isoenzyme MB (CK-MB) activity levels in serum, a marker of myocardial cell damage [16], [17], and examined ECG registers looking for electrical abnormalities [7], [18]. At 30 dpi, increased CK-MB activity was observed in *T. cruzi*-infected C57BL/6 mice when compared with their noninfected counterparts (**Figure 1C**). The CK-MB activity reached its highest levels at 90 dpi and remained high during chronic infection, which is suggestive of continuous cardiomyocyte injury (**Figure 1C**). Although during the acute infection there is a parallelism between cardiac parasitism and CK-MB activity in the serum (15–45 dpi, r^2^ = 0.982, *p*<0.001), no association between the intensity of cardiac parasitism and the evolution of cardiomyocyte lesion was noticed after parasite control or in chronic *T. cruzi* infection (60–120 dpi, r^2^ = 0.0399, *p*>0.05) (**Figure 1D**). Compared with sex- and age-matched noninfected controls, in infected mice, bradycardia was the first significant ECG alteration and was firstly detected at 30 dpi. During the course of the infection, the mice presented significantly higher bradycardia, PR intervals and prolonged QTc intervals when compared with noninfected controls (**Figure 1E** **and** **Table 1**). All (100%) of the infected mice presented ECG abnormalities at 90 dpi (**Table 1**) and featured a delay in the conduction of electric impulses together with arrhythmia and first- and second-degree atrioventricular blocks (AVB1 and AVB2). Again, there was no relationship between the intensity of tissue parasitism and the evolution of electrical abnormalities. Interestingly, the CK-MB activity in the serum and the electrical alterations were paralleled by heart enlargement during the chronic infection phase (**Figure 1F**). Therefore, we evaluated whether heart injury was associated with the establishment and the intensity of CD8-enriched myocarditis. A kinetic study of heart colonization by inflammatory CD4^+^ and CD8^+^ cells, the major components of chagasic myocarditis [5], [6], in *T. cruzi*-infected C57BL/6 mice revealed that rare mononuclear cells were detected in the cardiac tissue at 15 dpi. CD8-enriched inflammation occurred at 30 dpi, after which a significant contraction of inflammation was observed; however, myocarditis that was mainly composed of CD8^+^ T-cells persisted during the chronic phase (**Figure 1G**). Once again, there was no correlation between CK-MB activity levels in the serum and the intensity of inflammation (r^2^ = 0.038, *p*>0.05). Flow cytometry analysis of mononuclear cells harvested from cardiac tissue of *T. cruzi*-infected C57BL/6 mice confirmed the predominance of CD8^+^ T-cells (**Figure S1A**). A small part of the TCR^+^ cells were CD8^neg^CD4^+^ T-cells. Also, some CD8^neg^NK1.1^+^ cells were detected (**Figure S1B**). Further, CD8^+^ cells were mainly TCR^+^ cells at 40 dpi (**Figure S1C**). CD8^+^ T-cells-enriched myocarditis persisted at 90 dpi (R1 gate: 47.6–73.6% TCRαβ^+^: 17.3–19.4% TCR TCRαβ^+^CD4^+^ *vs.* 28.2–51% TCR TCRαβ^+^CD8^+^, two independent experiments) when the highest level of CK-MB activity in the serum, demarking the cardiomyocyte lesion (**Figure 1C**), was detected.

**Table 1:** Electrocardiograph parameters of C57BL/6 mice infected with the Colombian *T. cruzi* strain.

Groups	Heart rate^1^ (bpm)	PR interval (ms)	P duration (ms)	QRS duration (ms)	QTc (ms)	Cardiac conduction (% of mice)^2^	Frequency of afflicted mice (%)
Noninfected	637±70.7	35±1.1	9±2.1	11±1.5	77±5.7	ART (0%), AVB1 (0%), AVB2 (0%)	0
*T. cruzi* 30 dpi	491±36.2	41±2.8	13±1.2	13±2.3	81±5.1	ART (43%), AVB1 (43%), AVB2 (28%)	70
*T. cruzi* 60 dpi	496±51.3* ^,^ 3	46±4.2*	14±3.7	14±0.8	86±6.2	ART (57%), AVB1 (43%), AVB2 (43%)	86
*T. cruzi* 90 dpi	450±61.6**	47±6.3*	14±0.9	13±1.2	88±13.3*	ART (67%), AVB1(43%), AVB2 (57%)	100
*T. cruzi* 120 dpi	433±64.4***	45±4.7*	14±3.0	13±1.1	89±11.6**	ART (72%), AVB1 (28%), AVB2 (72%)	100

1 ECG parameters were evaluated using the following standard criteria: (i) heart rate (monitored by beats per minute (bpm), and (ii) the variation of the P wave and PR, QRS and corrected QT intervals (QTc), all measured in milliseconds (ms). ART, arrhythmia; AVB1, first-degree atrioventricular block; AVB2, second-degree atrioventricular block.

2 This data represent three independent experiments, with 7–8 infected mice/group. Three sex- and age-matched noninfected controls were analyzed in all experimental time points. The results were pooled in one representative group of 12 noninfected controls per experiment.

3 Significant differences

*: , *p*<0.05;

**: , *p*<0.01;

***: , *p*<0.001 between the values for noninfected and *T. cruzi*-infected groups of mice.

Therefore, the present findings show that chronic cardiomyopathy in C57BL/6 mice is not associated with the intensity of heart parasitism or inflammation, even though cardiomyocyte lesion and electrical abnormalities occurred in the persistence of the parasite and the CD8-enriched inflammation in this tissue. Collectively, these data show that this model reproduces several features of the CCC that has been described in patients [2] and it is an appropriate model for this study.

### Chronic heart injury was not related to the systemic immune response but paralleled the accumulation of anti-*T. cruzi* VNHRFTLV ASP2 effector CD8^+^ T-cells in the cardiac tissue

**Figure 2:**
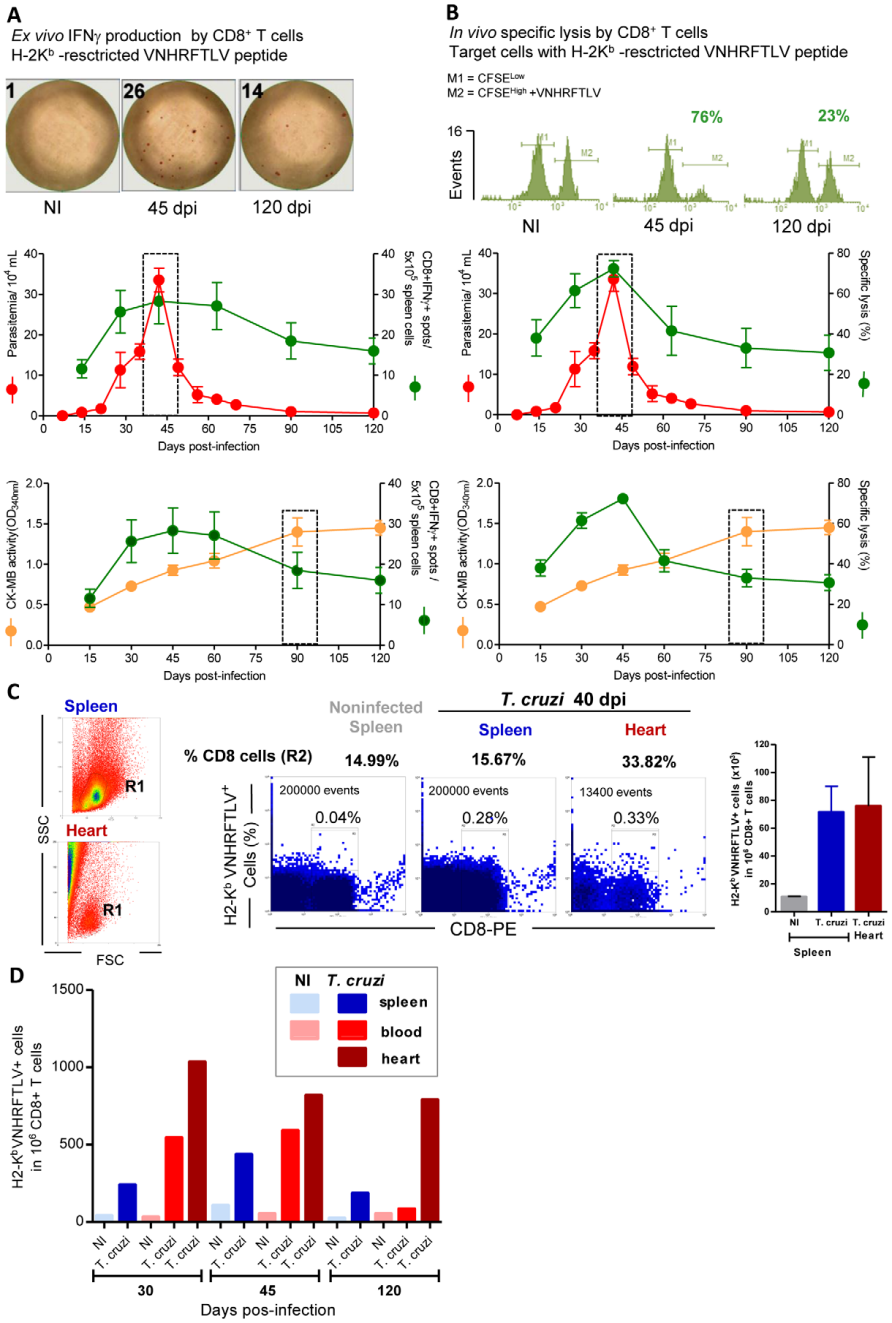
CD8^+^ T-cells recognizing the VNHRFTLV ASP2 *T. cruzi* peptide are enriched in the cardiac tissue. C57BL/6 mice were infected with 100 bt of the Colombian strain of *T. cruzi* and the anti-parasite immune response in the spleen and cardiac tissue was assessed. (**A**) Representative spots formed after stimulation of spleen cells from noninfected (NI) and *T. cruzi*-infected mice at 45 and 120 dpi with H-2K^b^-resctricted VNHRFTLV peptide. The number of CD8^+^IFNγ^+^ as determined by *ex vivo* ELISpot were analyzed and compared with parasitemia and CK-MB activity levels in the serum. (**B**) Representative histogram profiles of *in vivo* cytotoxicity assay showing the specific lysis of H-2K^b^-resctricted VNHRFTLV peptide-labeled CFSE^high^ cells from NI controls and *T. cruzi*-infected C57BL/6 mice at 45 and 120 dpi. The frequencies of *in vivo* specific lysis of with H-2K^b^ -resctricted VNHRFTLV peptide-labeled target cells in *T. cruzi*-infected C57BL/6 mice at 15, 30, 45, 60, 90 and 120 dpi were analyzed and compared with parasitemia and CK-MB activity levels in the serum. The peak of parasitemia and the maximum CK-MB activity are highlighted (dotted line). Each circle and vertical lines represent the mean ± standard deviation (SD) of the studied group (5–7 animals/time point). These data represent three independent experiments. (**C**) Frequency of double-positive CD8^+^ H-2K^b^ /VNHRFTLV^+^ cells [R1 (SSCxFSC) gated] of spleen and heart of NI and *T. cruzi*-infected C57BL/6 mice at 40 dpi. (**D**) Frequencies of double-positive CD8^+^ H-2K^b^ /VNHRFTLV^+^ cells [R1 (SSCxFSC) gated] of spleen, peripheral blood and heart of NI and *T. cruzi*-infected C57BL/6 mice at 30, 45 and 120 dpi. Representative flow ctometry profiles and mean ± SD of two or three animals per group. Bars represent the mean of two or three pools of 5 mice per pool.

Next, we investigated whether the induction of the anti-*T. cruzi* CD8-mediated immune response involving IFNγ production and the cytotoxic machinery was related to heart injury. In spleen of *T. cruzi*-infected mice, the *ex vivo* H-2K^b^-restricted anti-VNHRFTLV ASP2 peptide [19], [20] IFNγ-secreting cells (**Figure 2A**) and *in vivo* cytotoxic CD8^+^ T-cells (**Figure 2B**) were first detected at 15 dpi. Both of these CD8^+^ T-cell effector activities rose quickly, reaching a maximum at the parasitemia peak (45 dpi) and remaining high until 60 dpi. Both of the anti-VNHRFTLV CD8^+^ T-cell effector activities declined slowly thereafter, but they persisted after parasite control and remained detectable at 120 dpi (**Figure 2A and 2B**). Thus, during the acute phase (from 15 to 45 dpi) the increase of both CD8^+^ T-cell effector activities (inflammatory and cytolytic) in the spleen paralleled the parasitic load in the blood and in cardiac tissues. At 60 dpi, after the parasitemia and heart parasitism were under control (**Figure 2A and 2B**), there was contraction of these CD8^+^ T-cell effector activities, as expected. However, anti-VNHRFTLV CD8^+^ T-cell effector activities (inflammatory and cytolytic) persisted during the chronic infection and were associated with an increased spleen weight and cellularity (**Figure S2A and S2B**), although these effector activities were not proportional to the parasite load (**Figure 2A and 2B**). Moreover, during the chronic infection, no relationship was observed between the intensity of the systemic anti-VNHRFTLV CD8^+^ T-cell effector activities and cardiomyocyte lesion, as evidenced by the observation that the highest CK-MB activity level in the serum was detected at 90 dpi, which follows the contraction of CD8^+^ T-cell effector activities (inflammatory and cytolytic) (**Figure 2A and 2B**). Importantly, using the major histocompatibility complex class I (MHC I) multimer H-2K^b^/VNHRFTLV, a critical reagent for proper visualization of specific CD8^+^ T-cells [21], we revealed the presence of anti-VNHRFTLV ASP2 peptide CD8^+^ T-cells in spleen and cardiac tissue of the Colombian-infected C57BL/6 mice (**Figure 2C**). The analysis of CD8^+^ H-2K^b^/VNHRFTLV^+^ T-cells in spleen (**Figure 2D**) confirmed the kinetic of the frequency of anti-VNHRFTLV CD8^+^ T-cells in this tissue detected by *ex vivo* ELISpot and *in vivo* cytolytic assay. Further, the frequency of blood circulating CD8^+^ H-2K^b^/VNHRFTLV^+^ T-cells reflected the high frequency of these cells in the spleen in the acute infection and the contraction of these populations in the chronic infection (**Figure 2D**). Conversely, there was an enrichment in CD8^+^ H-2K^b^/VNHRFTLV^+^ T-cells among heart infiltrating inflammatory cells in the acute infection that persisted during the chronic phase of infection (**Figure 2D**), paralleling the persisting myocardial cell injury (**Figure 1C**).

### Heart injury is associated with the enrichment of Pfn^+^ cells in the cardiac tissue during the chronic *T. cruzi* infection

**Figure 3:**
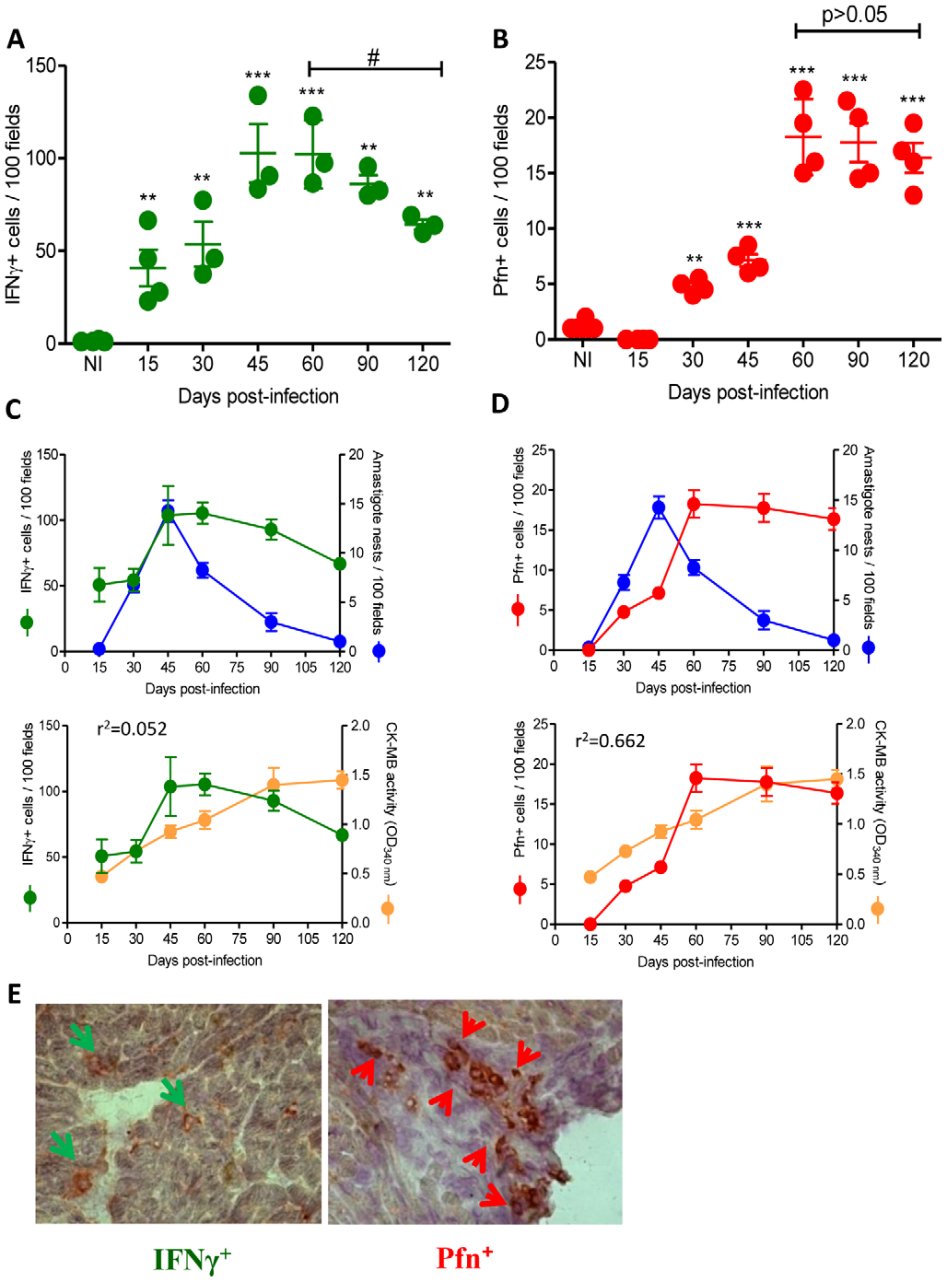
IFNγ^+^ and Pfn^+^ cells differentially invade the cardiac tissue of *T. cruzi*-infected C57BL/6 mice. Mice were infected with 100 bt of the Colombian strain of *T. cruzi* and the presence of IFNγ^+^ and Pfn^+^ cells in the cardiac tissue was evaluated by IHS. (**A**) Numbers of IFNγ^+^ cells infiltrating the cardiac tissue at 15, 30, 45, 60, 90 and 120 dpi. (**B**) Numbers of Pfn^+^ cells infiltrating the cardiac tissue at 15, 30, 45, 60, 90 and 120 dpi. (**C**) Relationship between IFNγ^+^ cells infiltrating the cardiac tissue and heart parasitism or CK-MB activity in serum. (**D**) Relationship between Pfn^+^ cells infiltrating the cardiac tissue and heart parasitism or CK-MB activity in the serum. (**E**) Immunohistochemistry staining of IFNγ^+^ (green arrows) and Pfn^+^ (red arrows) cells infiltrating the cardiac tissue at 60 dpi. In (**A**) and (**B**), each circle represents an individual mouse. In (**C**) and (**D**), each circle represents the mean ± SD of the studied group (5–7 animals/time point). These data represent three independent experiments. ^*^, *p*<0.05; ^**^, *p*<0.01; and ^***^, *p*<0.001, comparing noninfected (NI) controls and *T. cruzi*-infected mice. ^#^, *p*<0.05, comparing *T. cruzi*-infected mice at 60 and 120 dpi.

As described above, IHS and flow cytometry analysis showed that CD8-enriched myocarditis was established early during the *T. cruzi* infection. Initially, we adopted an *in situ* IHS approach to investigate whether inflammatory (IFNγ^+^) and cytotoxic (Pfn^+^) cells differentially colonize the cardiac tissue at different time points during *T. cruzi* infection. We evaluated the number of Pfn^+^ and IFNγ^+^ cells within the myocardium of C57BL/6 mice at 15, 30, 60, 90 and 120 dpi and revealed that IFNγ^+^ cells are detected at 15 dpi (**Figure 3A**), preceding the appearance of Pfn^+^ cells, which were first detected at 30 dpi (**Figure 3B**). Furthermore, the highest number of IFNγ^+^ cells was observed at 45 dpi, which coincides with the heart parasitism peak (**Figure 3C**), whereas the number of Pfn^+^ cells peaked at 60 dpi (**Figure 3D**). At 120 dpi, there was a significant (*p*<0.05) reduction in the number of IFNγ^+^ cells infiltrating the cardiac tissue, whereas the Pfn^+^ cell number remained high (**Figure 3A and 3B**). Therefore, the ratio of IFNγ^+^ to Pfn^+^ cells decreased and resulted in a relative enrichment of Pfn^+^ cells among the inflammatory cells that invaded the cardiac tissue as the infection approached the chronic phase (**Table 2**). Next, we determined whether the control of the parasite and the cardiomyocyte lesion were in any way related to the presence of IFNγ^+^ and Pfn^+^ cells in the cardiac tissue in response to *T. cruzi* infection. The analysis of the IHS data showed that the heart parasitism was under control only after 60 dpi when both Pfn^+^ and IFNγ^+^ cells were detected in high numbers in the cardiac tissue (**Figure 3C and 3D**). Importantly, in the chronic infection cardiomyocyte lesion was not related to the presence of IFNγ^+^ cells (r^2^ = 0.052), but correlated with the persistence of high number of Pfn^+^ cells (r^2^ = 0.662) invading the cardiac tissue (**Figure 3C and 3D**). Interestingly, the distribution of Pfn^+^ cells in the cardiac tissue was more focal, whereas the IFNγ^+^ cells were spread throughout the myocardium (**Figure 3E**). Taken together, these data suggest a role for the infiltrating inflammatory Pfn^+^ cells in heart injury during *T. cruzi* infection.

**Table 2:** Number of IFNγ^+^ and Pfn^+^ cells in heart tissue sections of *T. cruzi*-infected C57BL/6 mice.

dpi^1^	IFNγ^+^ cells /100 fields^2^	Pfn^+^ cells /100 fields	Ratio of IFNγ^+^/Pfn^+^ cells^3^
15	50±28	0	50∶0
30	54±17	5±1	11∶1
45	103±45	7±1	14∶1
60	105±16	18±3	5∶1
90	92±15	18±3	5∶1
120	66±7	17±3	4∶1

1 dpi, days post-infection with 100 bt of the Colombian *T. cruzi* strain.

2 The number of positive cells for each parameter was counted in 100 microscopic fields per section. Three sections were counted per each analyzed animal.

3 Ratio of IFNγ^+^/Pfn^+^ cells was determined adopting the mean number of positive cells for each parameter per 100 microscopic fields.

### Segregation of CD8^+^ T-cells into IFNγ^+^Pfn^neg^ and IFNγ^neg^Pfn^+^ populations in *T. cruzi* infection

**Figure 4:**
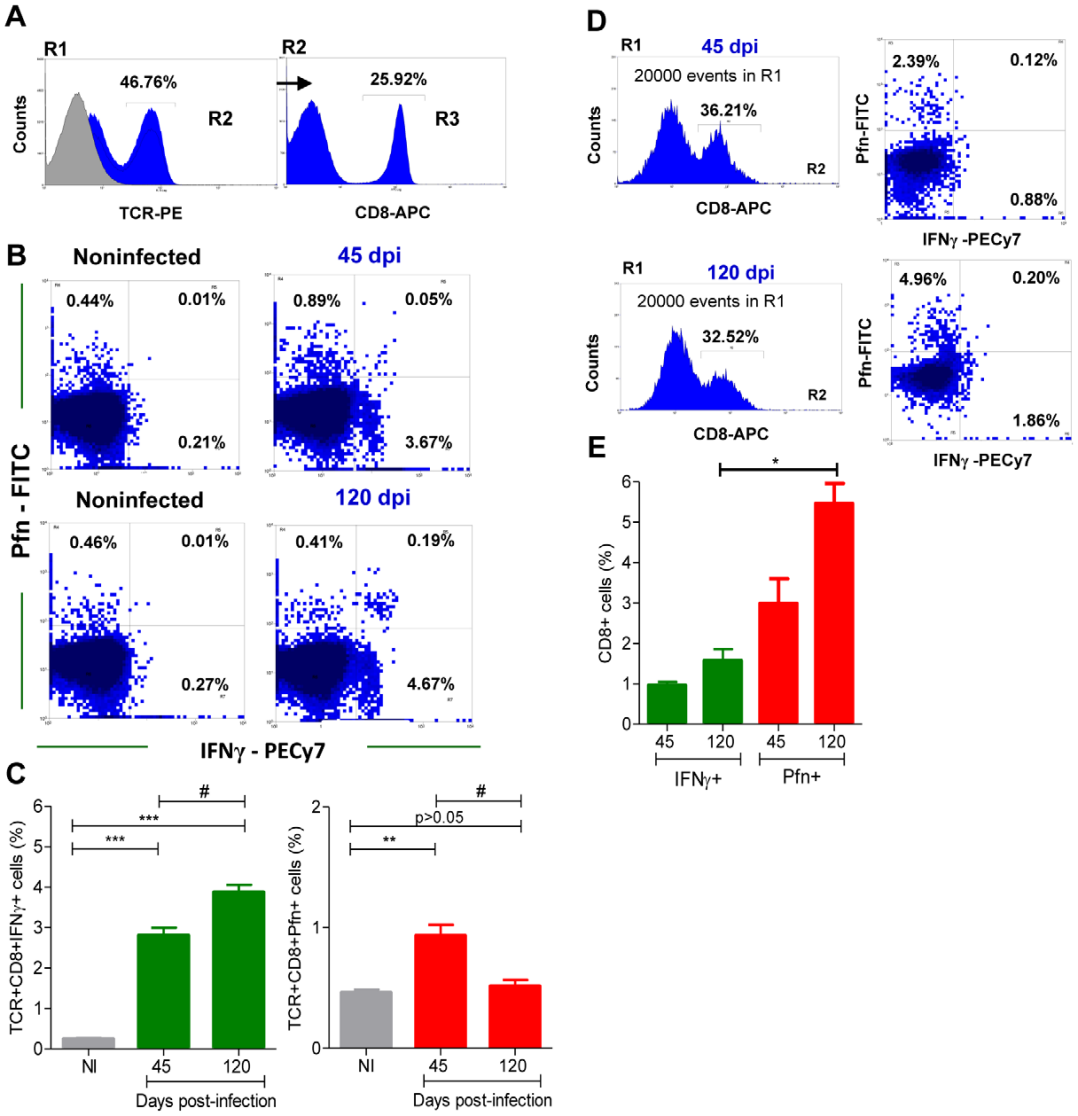
Segregation of CD8^+^ T-cells into IFNγ^+^ and Pfn^+^ populations in *T. cruzi* infection. C57BL/6 mice were infected with 100 blood trypomastigotes of the Colombian strain of *T. cruzi* and the expression of IFNγ^+^ and Pfn^+^ by CD8^+^ cells in the peripheral blood and cardiac tissue was evaluated. (**A**) Representative histograms of flow cytometry analysis of peripheral blood CD8^+^ T-cells [R1 (SSCxFSC)/R2 (TCR)/R3 (CD8) gated] that were analyzed for IFNγ and Pfn expression in *T. cruzi*-infected mice (120 dpi). (**B**) Representative dot plots of flow cytometry analysis of peripheral blood CD8^+^ T-cells (R1/R2/R3 gated) expressing IFNγ and Pfn at 45 and 120 dpi. (**C**) Frequency of double-stained CD8^+^IFNγ^+^ and CD8^+^Pfn^+^ peripheral blood T-cells cells (R1/R2/R3 gated) at 45 and 120 dpi. (**D**) Representative histograms and dot plots of flow cytometry analysis of heart infiltrating CD8^+^ cells [R1 (SSCxFSC)/R2 (CD8) gated] expressing IFNγ and Pfn at 45 and 120 dpi. (**E**) Frequency of double-stained CD8^+^IFNγ^+^ and CD8^+^Pfn^+^ of heart infiltrating cells (R1/R2 gated) at 45 and 120 dpi. In (**C**) Bars represent the mean ± SD of five to eight mice per group. In (**E**) Bars represent the mean of two or three pools of 5 mice per group. These data represent two or three independent experiments. ^*^, *p*<0.05; ^**^, *p*<0.01; and ^***^, *p*<0.001, comparing noninfected (NI) controls and *T. cruzi*-infected mice. ^#^ *P*<0.05, comparing *T. cruzi*-infected mice at 45 and 120 dpi.

The kinetics of cardiac tissue colonization by IFNγ^+^ and Pfn^+^ cells suggest that the inflammatory and cytotoxic effector activities were segregated into different cell populations. Therefore, we investigated whether CD8^+^ T-cells express IFNγ and Pfn in a multifunctional or segregated manner during *T. cruzi* infection. Peripheral blood CD8^+^ T-cells (**Figure 4A**), which potentially migrate to the cardiac tissue, from acute and chronically *T. cruzi*-infected C57BL/6 mice were assessed for intracellular IFNγ and Pfn expression. In comparison with noninfected mice, there was a significant increase in the frequency of CD8^+^ T-cells expressing IFNγ and Pfn in the peripheral blood of *T. cruzi*-infected mice (**Figure 4B and 4 C**). Indeed, most of the CD8^+^ T-cells segregated into CD8^+^IFNγ^+^Pfn^neg^ (CD8^+^IFNγ^+^) and CD8^+^IFNγ^neg^Pfn^+^ (CD8^+^Pfn^+^) cell populations (**Figure 4B**). In the circulating blood, there was a predominance of CD8^+^IFNγ^+^ cells in comparison with CD8^+^Pfn^+^ cells during the acute (45 dpi) and chronic (120 dpi) phases of the infection (**Figure 4B and 4C**). CD8^+^IFNγ^+^Pfn^+^ cells were barely detected in noninfected and *T. cruzi*-infected mice, although a careful analysis revealed an upregulation of this rare CD8^+^ T-cell population during the chronic infection (from 0.01–0.05% in noninfected to 0.19–0.34% in chronically infected mice). Interestingly, during the chronic infection there was an accumulation of CD8^+^IFNγ^+^ cells in the peripheral blood, whereas a decrease in the frequency of CD8^+^Pfn^+^ cells was detected (**Figure 4B and 4C**). Collectively, these data suggest that in the peripheral blood most of the CD8^+^ T-cell effectors potentially able to perform inflammatory and cytotoxic activities are phenotypically segregated, respectively, into CD8^+^IFNγ^+^ and CD8^+^Pfn^+^ T-cells during the acute and chronic phases of *T. cruzi* infection. Importantly, this segregation of CD8^+^ T-cells into CD8^+^IFNγ^+^ and CD8^+^Pfn^+^ populations was also detected in the inflammatory cells infiltrating the cardiac tissue at 45 and 120 dpi (**Figure 4D**). In addition, the cardiac tissue infiltrating CD8^+^IFNγ^+^ cells were IFNγ^dull^ (MFI = 33.4–38.66) when compared with the CD8^+^IFNγ^+^ cells in peripheral blood (MFI = 144.12–166.81). Moreover, in the cardiac tissue there was prevalence of CD8^+^Pfn^+^ cells in the acute and, particularly, in the chronic *T. cruzi* infection (**Figure 4D and 4E**).

Considering the recent finding showing a role for interleukin (IL)-10 in *T. cruzi*-triggered myocarditis [22], we studied whether or not CD8^+^IFNγ^+^ cells coexpress IL-10 during *T. cruzi* infection. The analysis of CD8^+^ T-cells in the peripheral blood revealed that most of the IFNγ^+^ cells were CD8^+^IFNγ^+^IL-10^+^ in the acute and in the chronic infection (**Figure S3A**) and accumulated in blood in the chronic phase of infection (**Figure S3B**). Further, there was a significant frequency of CD8^+^IFNγ^+^IL-10^neg^ T-cells accumulating in the peripheral blood in the chronic infection (**Figure S3C)**. More important, in the heart tissue most of the CD8^+^IFNγ^dull^ cells do not express IL-10 (**Figure S3D**) or express this cytokine in a very transient manner and were not detected in our experimental approach.

### Differential compartmentalization of anti- *T. cruzi* CD8^+^ T-cells expressing IFNγ and Pfn

**Figure 5:**
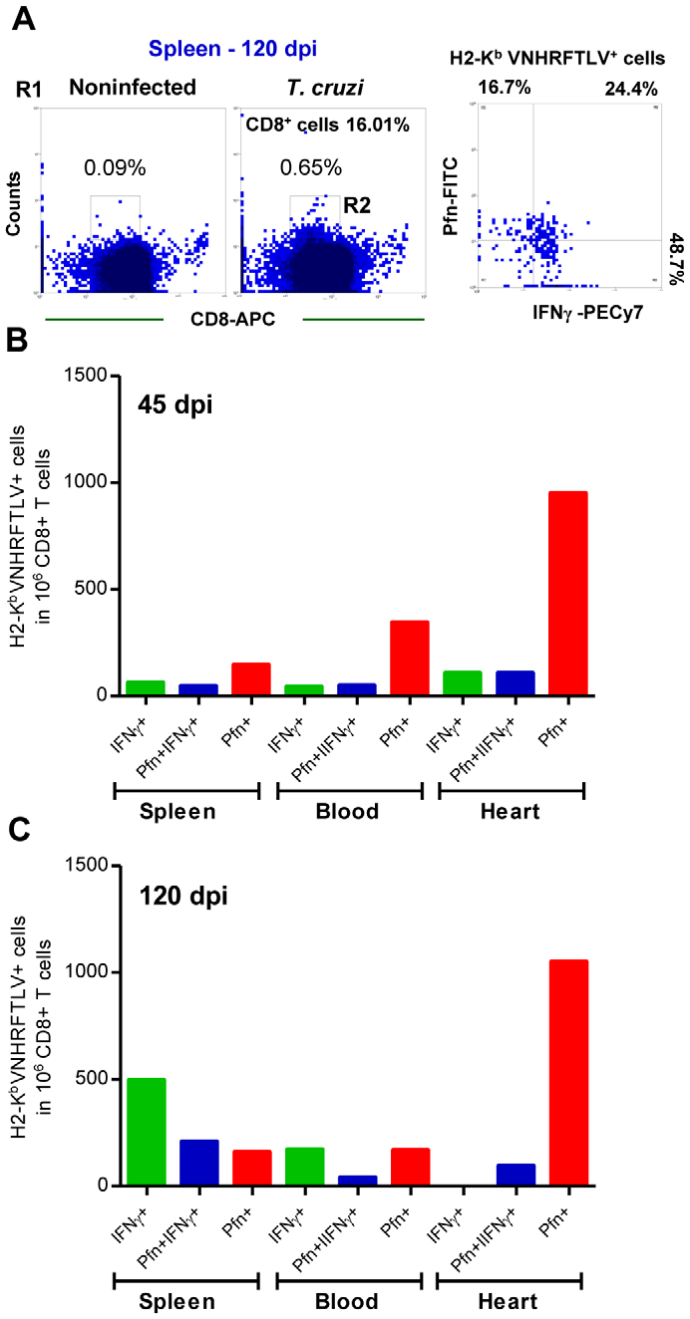
Differential compartmentalization of anti-*T. cruzi* CD8^+^ T-cells expressing IFNγ and Pfn. C57BL/6 mice were infected with 100 bt of the Colombian strain of *T. cruzi* and presence of CD8^+^ H-2K^b^ /VNHRFTLV^+^ T-cells expressing IFNγ^+^ and Pfn^+^ in the spleen, peripheral blood and in the cardiac tissue was evaluated. (**A**) Representative dot plots of double-positive CD8^+^ H-2K^b^ /VNHRFTLV^+^ T-cells (R1 gated) in the spleen of NI and *T. cruzi*-infected C57BL/6 mice at 120 dpi. Frequencies of CD8^+^ H-2K^b^ /VNHRFTLV^+^ T-cells (R2 gated) expressing IFNγ, Pfn or coexpressing IFNγ and Pfn in the spleen, peripheral blood and cardiac tissue of NI and *T. cruzi*-infected C57BL/6 mice at 45 (**B**) and 120 dpi (**C**). Bars represent the means of two or three pools of 5 mice per group. These data represent two independent experiments.

The accumulation of CD8^+^IFNγ^+^ cells in the peripheral blood and the enrichment in CD8^+^Pfn^+^ cells in the cardiac tissue during the chronic *T. cruzi* infection led us to investigate whether or not there was distinct distribution of the CD8^+^ H-2K^b^/VNHRFTLV^+^ T-cells expressing IFNγ and Pfn in different immune compartments of an infected mice. Indeed, in the spleen there was a predominance of H-2K^b^/VNHRFTLV^+^ IFNγ^+^ cells, followed by double-positive IFNγ^+^Pfn^+^ cells and Pfn^+^ cells at 120 dpi (**Figure 5A**). Therefore, we analyzed the presence of CD8^+^ H-2K^b^/VNHRFTLV^+^ cells expressing IFNγ and Pfn in the spleen, peripheral blood and cardiac tissue during the acute and chronic infection of C57BL/6 mice. In the acute infection (45 dpi), there was predominance of H-2K^b^/VNHRFTLV^+^ Pfn^+^ cells in the spleen, peripheral blood and cardiac tissue inflammatory CD8^+^ T-cells (**Figure 5B**). Moreover, in the chronic phase of infection (120 dpi) the accumulation of CD8^+^ H-2K^b^/VNHRFTLV^+^ IFNγ^+^ cells in spleen was confirmed. Anti-*T. cruzi* IFNγ^+^Pfn^+^ cells were also retained in the spleen at 120 dpi (**Figure 5C**). In contrast, similar frequencies of segregated CD8^+^ H-2K^b^/VNHRFTLV^+^ IFNγ^+^ or Pfn^+^ cells were detected in peripheral blood, while the enrichment in CD8^+^ H-2K^b^/VNHRFTLV^+^ Pfn^+^ cells among heart infiltrating inflammatory cells persisted at 120 dpi (**Figure 5C**), paralleling the myocardial cell injury (**Figure 1C**).

### CD8^+^IFNγ^+^ and CD8^+^Pfn^+^ cells differentially express CCR5 and LFA-1

Our findings showed differential accumulation of CD8^+^IFNγ^+^ cells in the peripheral blood and CD8^+^Pfn^+^ T-cells in the cardiac tissue during the chronic *T. cruzi* infection (**Figure 4**). Based on these findings and on previous data demonstrating that peripheral blood CD8^+^CCR5^+^LFA-1^+^ cells drastically increase during *T. cruzi* infection [12], we next examined whether CCR5 and LFA-1, molecules essential for the migration of inflammatory cells towards the cardiac tissue during *T. cruzi* infection [11], [12], [23], were differentially expressed by the CD8^+^IFNγ^+^ and CD8^+^Pfn^+^ cells. For that, we analyzed the frequencies of CCR5^+^LFA-1^+^ cells among CD8^+^IFNγ^+^ (**Figure S4**) and CD8^+^Pfn^+^ peripheral blood cells. A low proportion of peripheral blood CD8^+^IFNγ^+^ cells were CCR5^+^LFA-1^+^, whereas a high proportion of CD8^+^Pfn^+^ cells were CCR5^+^LFA-1^+^ during the acute (45 dpi) and chronic (120 dpi) phases of infection (**Table 3**). Therefore, although present at a lower frequency than CD8^+^IFNγ^+^ cells in peripheral blood, a higher proportion of CD8^+^Pfn^+^ cells coexpress CCR5 and LFA-1, and they are potentially more prone to migrate than CD8^+^IFNγ^+^ cells.

**Table 3:** Frequency of CD8^+^IFNγ^+^ and CD8^+^Pfn^+^ coexpressing CCR5 and LFA-1 among peripheral blood cells during acute and chronic *T. cruzi* infection.

Groups	CD8^+^IFNγ^+^ CCR5^+^LFA-1^+^ (%)	CD8^+^Pfn^+^ CCR5^+^LFA-1^+^ (%)
Noninfected	1.00±0.27	1.01±0.64
42 dpi (acute)	34.52±8.98	53.12±5.64
120 dpi (chronic)	1.01±0.64	6.30±0.38

### Distinct migratory behavior of CD8^+^cells from *ifnγ* ^+/+^ *pfn* ^−/−^ and *ifnγ* ^−/−^ *pfn* ^+/+^ mice towards cardiac tissue during *T. cruzi* infection

**Figure 6:**
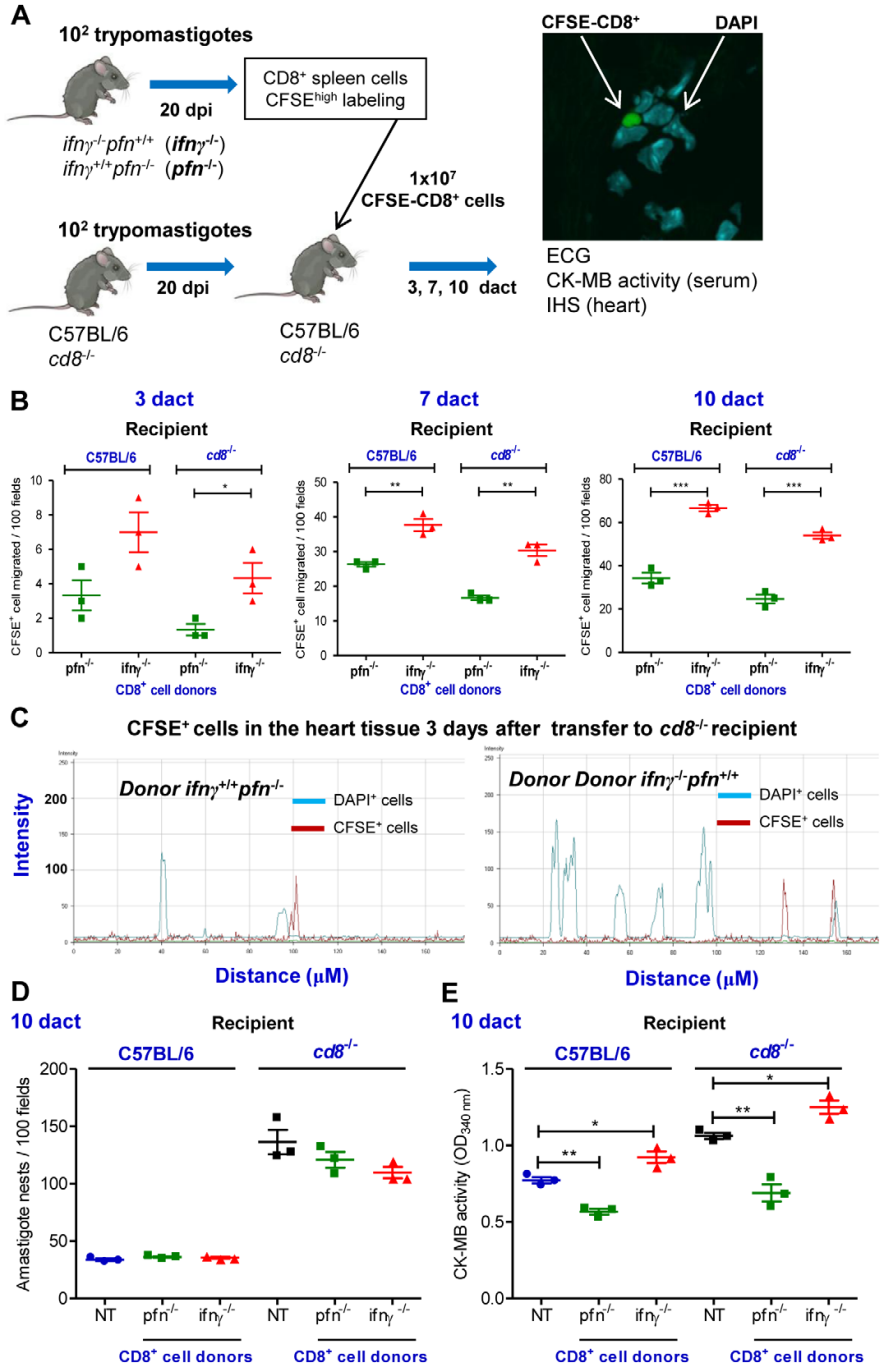
CD8^+^ cells from *ifnγ* ^−/−^ *pfn* ^+/+^ and *ifnγ* ^+/+^ *pfn* ^−/−^ infected mice differentially migrate to the cardiac tissue. (**A**) Experimental scheme showing the infection of donors and recipients mice by *T. cruzi*, isolation of CD8^+^ cells from *ifnγ* ^−/−^ *pfn* ^+/+^ and *ifnγ* ^+/+^ *pfn* ^−/−^ infected donors (20 dpi) by magnetic beads, CFSE^high^ labeling, adoptive transfer of CFSE^+^CD8^+^ cells to C57BL/6 and *cd8* ^−/−^ infected recipients (20 dpi) and analysis at 3, 7 and 10 days after cell transfer (dact). The colonization of the cardiac tissue by CFSE^+^ cells, heart parasitism and CK-MB activity levels in the serum were evaluated. (**B**) Detection of CFSE^+^CD8^+^ cells in cardiac tissue sections at 3, 7 and 10 dact to *T. cruzi*-infected C57BL/6 and *cd8* ^−/−^ recipients. (**C**) Graphics showing the confocal analysis of the intensity of fluorescence of CFSE^+^CD8^+^ cells present in the myocardium of cardiac tissue sections at 3 dact to *T. cruzi*-infected *cd8* ^−/−^ recipients. DAPI was used to reveal the nucleus of the cells of the recipient mice. (**D**) Number of amastigote nests in 100 microscopic fields of cardiac tissue from C57BL/6 and *cd8* ^−/−^ infected mice that were non-transferred (NT) or transferred with CD8^+^ cells from *ifnγ* ^−/−^ *pfn* ^+/+^ and *ifnγ* ^+/+^ *pfn* ^−/−^ donors, at 10 dact. (**E**) Cardiomyocyte lesions were evaluated at 10 dact by measuring the CK-MB activity in serum samples from C57BL/6 and *cd8* ^−/−^ infected mice that were non-transferred (NT) or transferred with CD8^+^ cells from *ifnδ* ^−/−^ *pfn* ^+/+^ and *ifnγ* ^+/+^ *pfn* ^−/−^ donors. Each symbol represents an individual mouse. These data represent three independent experiments. ^*^, *p*<0.05; ^**^, *p*<0.01; and ^***^, *p*<0.001, comparing C57BL/6 and *cd8* ^−/−^ infected mice that were non-transferred and transferred with CD8^+^ cells from *ifnγ* ^−/−^ *pfn* ^+/+^ and *ifnγ* ^+/+^ *pfn* ^−/−^ donors.

To investigate whether CD8^+^IFNγ^+^ and CD8^+^Pfn^+^ cells exhibit a distinct migratory behavior with a differential potential to colonize the cardiac tissue, a series of adoptive cell transfer experiments was performed. In brief, *ifnγ* ^+/+^ *pfn* ^−/−^ and *ifnγ* ^−/−^ *pfn* ^+/+^ mice were infected and their spleens were removed at 20 dpi (100% of the animals were alive at this time point). The *T. cruzi*-infected *ifnγ* ^+/+^ *pfn* ^−/−^ and *ifnγ* ^−/−^ *pfn* ^+/+^ mice presented increased spleen cellularity, similar to infected C57BL/6 mice (**Figure S5A**). These infected *ifnγ* ^+/+^ *pfn* ^−/−^ and *ifnγ* ^−/−^ *pfn* ^+/+^ mice presented similar frequency of TCR^+^ cells, but infected *ifnγ* ^+/+^ *pfn* ^−/−^ mice had higher frequency of CD8^+^ T-cells (**Figure S5A**). The CD8^+^ T-cells from infected *ifnγ* ^+/+^ *pfn* ^−/−^ mice expressed IFNγ, whereas CD8^+^ T-cells from infected *ifnγ* ^−/−^ *pfn* ^+/+^ mice expressed Pfn (**Figure S5B**). Further, CD8^+^ T-cells from infected *ifnγ* ^+/+^ *pfn* ^−/−^ and *ifnγ* ^−/−^ *pfn* ^+/+^ mice were potentially functional as they expressed activation markers as IL-10, TNF and CD107a (**Figure S5B**). Therefore, single-cell suspensions of CD8-enriched cells (from infected *ifnγ* ^−/−^ *pfn* ^+/+^ or *ifnγ* ^+/+^ *pfn* ^−/−^ donors) were prepared using magnetic beads, labeled with CFSE and intravenously transferred into *cd8* ^−/−^ mice and C57BL/6 mice that had been infected with *T. cruzi* 20 days before. At 3, 7 and 10 days after the cell transfer (dact) (i.e., 23, 27 and 30 dpi; all of the recipient mice were alive) the hearts were removed from the *cd8* ^−/−^ and C57BL/6 recipient mice and the CFSE^+^CD8^+^ T-cells were examined and counted under fluorescence and confocal microscopes (**Figure 6A**). Our data indicate that more CFSE^+^CD8^+^ cells accumulated in the cardiac tissue of the *cd8* ^−/−^ mice that had received CFSE^+^CD8-enriched cells from *ifnγ* ^−/−^ *pfn* ^+/+^ donors compared with *cd8* ^−/−^ mice that had received CFSE^+^CD8-enriched cells from *ifnγ* ^+/+^ *pfn* ^−/−^ donors at all of the assessed time points (**Figure 6B**). Although the immunocompetent C57BL/6 recipient mice that had received CD8^+^ cells from *ifnγ* ^+/+^ *pfn* ^−/−^ or *ifnγ* ^−/−^ *pfn* ^+/+^donors accumulated more CFSE^+^CD8^+^cells among the inflammatory cells infiltrating the cardiac tissue compared with the *cd8* ^−/−^ recipients, the preferential accumulation of CFSE^+^CD8^+^ cells from *ifnγ* ^−/−^ *pfn* ^+/+^ donors was reproduced (**Figure 6B**). The prevalence of CFSE^+^CD8^+^ cells from *ifnγ* ^+/+^ *pfn* ^−/−^ donors in the cardiac tissue of recipient mice supports distinct migratory behavior of these cells rather than differential cell proliferation. Indeed, the similar intensities of fluorescence detected in the CFSE^+^CD8^+^ cells from *ifnγ* ^+/+^ *pfn* ^−/−^ and *ifnγ* ^−/−^ *pfn* ^+/+^ donors infiltrating the cardiac tissues of recipients at 3 dact supports that, at least in the initial days after cell transfer, there was no differential activation of the transferred CFSE^+^CD8^+^ cells (**Figure 6C**). Moreover, splenocytes from *T. cruzi*-infected mice, independently of the mouse lineage, were unresponsive to *in vitro* activation with anti-CD3/anti-CD28 stimuli (**Figure S5C**), while splenocytes isolated from naïve *ifnγ* ^+/+^ *pfn* ^−/−^mice were more responsive to anti-CD3/anti-CD28 stimuli (**Figure S5C**), particularly the CD8^+^ T-cells (**Figure S5D**). Interestingly, similar to what was detected in *T. cruzi*-infected C57BL/6 mice, the CFSE^+^CD8^+^ cells from *ifnγ* ^+/+^ *pfn* ^−/−^ donors were diffusely spread in the myocardium tissue, while the CFSE^+^CD8^+^ cells from *ifnγ* ^−/−^ *pfn* ^+/+^ donors were focally localized among inflammatory cells of the recipient (**Figure 6C**). Thus, CD8^+^IFNγ^neg^ (potentially Pfn^+^) cells migrated and accumulated in the cardiac tissue more readily than the CD8^+^Pfn^neg^ (potentially IFNγ^+^) cells during *T. cruzi* infection.

Next, we determined whether the differential colonization of the cardiac tissue of *T. cruzi*-infected C57BL/6 and *cd8* ^−/−^ recipient mice by CD8^+^ T-cells from *ifnγ* ^−/−^ *pfn* ^+/+^ and *ifnγ* ^+/+^ *pfn* ^−/−^ donors had a differential impact on parasite dissemination control and cardiomyocyte lesion. At 10 dact, similar cardiac tissue parasitism was observed in recipient mice that had received CFSE^+^CD8^+^ cells from *ifnγ* ^+/+^ *pfn* ^−/−^ or *ifnγ* ^−/−^ *pfn* ^+/+^ donors (**Figure 6D**). In C57BL/6 and *cd8* ^−/−^ recipient mice, the CFSE^+^CD8^+^ cells from *ifnγ* ^+/+^ *pfn* ^−/−^ donors had a beneficial impact on cardiomyocyte lesion as evidenced by a decrease in CK-MB activity levels in the serum compared with non-transferred (NT) mice. In contrast, the CFSE^+^CD8^+^ cells from *ifnγ* ^−/−^ *pfn* ^+/+^ donors had a detrimental impact as evidenced by the increase in CK-MB activity levels in the serum compared with their respective NT counterparts (**Figure 6E**). Therefore, these data circumstantially support that CD8^+^IFNγ^neg^ (potentially Pfn^+^) cells play a non-beneficial role in heart injury during *T. cruzi* infection.

### Colonization of the cardiac tissue with CD8^+^Pfn^+^ T-cells was correlated with cardiomyocyte lesion

**Figure 7:**
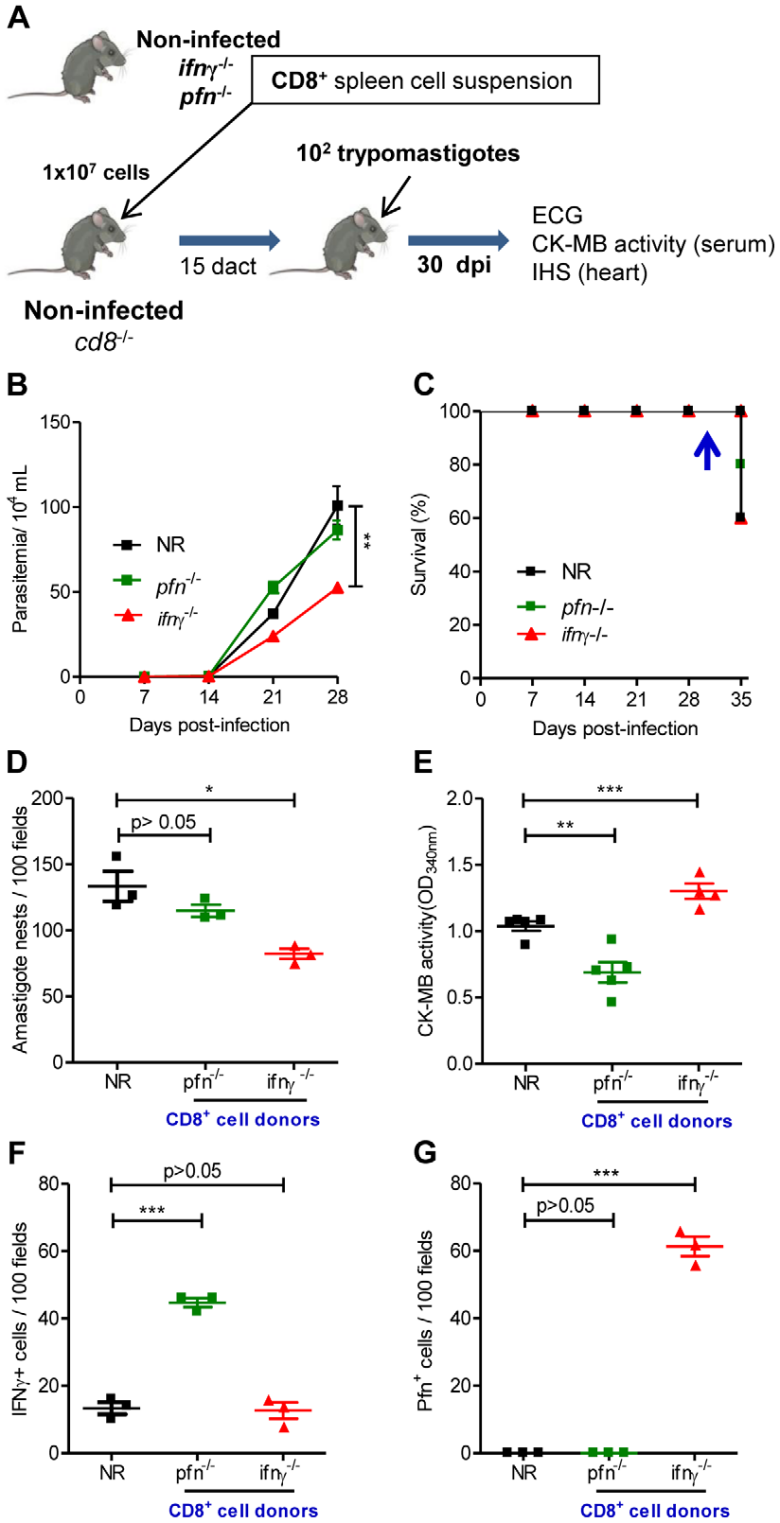
Distinct migratory behavior and effector function of CD8^+^IFNγ^+^ and CD8^+^Pfn^+^ T-cells in *T. cruzi* infection. (**A**) Experimental scheme of CD8^+^ cell isolation from noninfected naïve *ifnγ* ^−/−^ *pfn* ^+/+^ and *ifnγ* ^+/+^ *pfn* ^−/−^ donors, *in vivo* reconstitution of the CD8^+^ cell compartment of naïve *cd8* ^−/−^ mice, infection with 100 bt of the Colombian strain at 15 days after cell transfer (dact) and analysis at 30 days post-infection. Parasitemia, survival rate, cardiac parasitism and CK-MB activity levels in the serum and colonization of the cardiac tissue by IFNγ^+^ and Pfn^+^ cells were evaluated. (**B**) Parasitemia and (**C**) survival curve of *cd8* ^−/−^ mice non-reconstituted (NR) or reconstituted with CD8^+^ cells from *ifnγ* ^+/+^ *pfn* ^−/−^ and *ifnγ* ^−/−^ *pfn* ^+/+^ donors. In independent experiments, the animals were analyzed at 30 dpi when 100% of the mice in all the experimental groups were alive (arrow). (**D**) Number of amastigote nests in 100 microscopic fields of cardiac tissue sections. (**E**) Cardiomyocyte lesion evaluated by CK-MB activity in serum samples. The number of (**F**) IFNγ^+^ and (**G**) Pfn^+^ cells in 100 microscopic fields of cardiac tissue sections from mice non-reconstituted (NR) or reconstituted with CD8^+^ cells from *ifnγ* ^+/+^ *pfn* ^−/−^ and *ifnγ* ^−/−^ *pfn* ^+/+^ donors, at 30 dpi. Each symbol represents an individual mouse. These data represent three independent experiments. ^*^, *p*<0.05; ^**^, *p*<0.01; and ^***^, *p*<0.001, comparing *cd8* ^−/−^ infected mice non-reconstituted and reconstituted with CD8^+^ cells from *ifnγ* ^−/−^ *pfn* ^+/+^ and *ifnγ* ^+/+^ *pfn* ^−/−^ donors.

Based on the data presented above, we speculate that CD8^+^IFNγ^+^ and CD8^+^Pfn^+^ T-cells play distinct roles in the *T. cruzi*-elicited cardiomyopathy. To assess this theory, we selectively reconstituted the CD8 compartment of *cd8* ^−/−^ mice. Briefly, the spleens were removed from noninfected naïve *ifnγ* ^−/−^ *pfn* ^+/+^ (with the potential to generate Pfn^+^ cells) and *ifnγ* ^+/+^ *pfn* ^−/−^ (with the potential to generate IFNγ^+^ cells) mice and single-cell suspensions of CD8-enriched T-cells were transferred to noninfected naïve recipient *cd8* ^−/−^ mice (**Figure 7A**). The analysis of the peripheral blood of naïve recipients showed the presence of circulating CD8^+^ cells (2 to 6.5% of peripheral blood cells) in *cd8* ^−/−^ mice reconstituted with CD8^+^ cells from naïve *ifnγ* ^−/−^ *pfn* ^+/+^ and *ifnγ* ^+/+^ *pfn* ^−/−^ donors in all analyzed recipient mice at 15 days after cell transfer (**Figure S6**), at which time non-reconstituted (NR) and CD8-reconstituted mice were infected with *T. cruzi* (**Figure 7A**). Parasitemia was monitored every two days and heart parasitism and injury were evaluated at 30 dpi (at which time all recipient mice from every experimental group were alive). As expected, when compared with NR *T. cruzi*-infected *cd8* ^−/−^ mice at 28 dpi, the reconstitution of the CD8 compartment with CD8^+^ cells from *ifnγ* ^−/−^ *pfn* ^+/+^ or *ifnγ* ^+/+^ *pfn* ^−/−^ donors prior to infection did not worsen parasitemia. Furthermore, *cd8* ^−/−^ mice that had been reconstituted with CD8^+^ T-cells from *ifnγ* ^−/−^ *pfn* ^+/+^ donors presented reduced parasitemia when compared with NR *cd8* ^−/−^ mice (**Figure 7B**). All of the animals in every experimental group survived until 34 dpi. At 35 dpi, the first deaths were recorded in all groups (**Figure 7C**). In independent experiments, cardiac tissue parasitism was analyzed at 30 dpi when 100% of the mice in all the experimental groups were alive, revealing that the non-reconstituted *cd8* ^−/−^ mice presented high parasitism, as expected (**Figure 7D**). The reconstitution of the *cd8* ^−/−^ mice with CD8^+^ cells from *ifnγ* ^+/+^ *pfn* ^−/−^ donors neither ameliorated nor aggravated heart parasitism, whereas the reconstitution of the *cd8* ^−/−^ mice with CD8^+^ cells from *ifnγ* ^−/−^ *pfn* ^+/+^ donors significantly reduced heart parasitism (**Figure 7D**). To evaluate the participation of the distinct CD8^+^ cells populations in heart injury, CK-MB activity levels in the serum and the ECG registers were evaluated. Compared with the NR *cd8* ^−/−^ animals, *cd8* ^−/−^ mice that were reconstituted with CD8^+^ cells from *ifnγ* ^+/+^ *pfn* ^−/−^ donors exhibited a significant decrease in CK-MB activity levels in the serum at 10 dact (**Figure 7E**). In accordance with these findings, the ECG registers revealed that the reconstitution of *cd8* ^−/−^ recipients of CD8^+^ T-cells from *ifnγ* ^+/+^ *pfn* ^−/−^ donors was beneficial, reducing the frequency of afflicted mice and the severity of the electrical abnormalities (**Table 4**). On the other hand, the *cd8* ^−/−^ mice that had been reconstituted with CD8^+^ cells from *ifnγ* ^−/−^ *pfn* ^+/+^ donors exhibited an aggravation of myocardial cell lesion when compared with NR *cd8* ^−/−^ mice at 10 dact (**Figure 7E**). Further, the electrical abnormalities observed in *cd8* ^−/−^ mice that had been reconstituted with CD8^+^ T-cells from *ifnγ* ^−/−^ *pfn* ^+/+^ donors prior to infection were similar to those detected in NR *cd8* ^−/−^ mice (**Table 4**).

**Table 4:** Electrocardiograph parameters of *cd8* ^−/−^ mice adoptively transferred with CD8^+^ cell from *ifnγ* ^−/−^ *pfn* ^+/+^ or *ifnγ* ^+/+^ *pfn* ^−/−^ and infected with the Colombian *T. cruzi* strain.

Groups^1^	Heart rate^2^ (bpm)	PR interval (ms)	P duration (ms)	QRS duration (ms)	QTc (ms)	Cardiac conduction (% of mice)^3^	Frequency of afflicted mice (%)
*cd8* ^−/−^ NI	522±67	35±5.9	12±1.9	11±2.1	70±11.3	ART (0%), AVB1(0%), AVB2 (0%)	0
*cd8* ^−/−^ (NR) *T. cruzi*	311±59* ^,^ 4	38±4.1	16±2.4*	14±1.3*	106±6.8*	ART (60%), AVB1 (0%), AVB2 (100%)	100
*cd8* ^−/−^AdT-CD8^+^ *pfn* ^−/−^ *T. cruzi*	465±80#	34±4.9	12±2.3#	13±2.6	88±11.0	ART (40%), AVB1 (20%), AVB2 (60%)	80
*cd8* ^−/−^AdT-CD8^+^ *ifnγ* ^−/−^ *T. cruzi*	341±97	42±2.6	15±3.2	16±7.1	109±15.8	ART (80%), AVB1 (0%), AVB2 (100%)	100

1 *cd8* ^−/−^ mice were: NI, non-infected; *T. cruzi*, infected with 100 bt forms of the Colombian *T. cruzi* strain 15 days after adoptive cell transfer (AdT) of CD8-enriched cells (≥98%), obtained from IFNγ-deficient (*ifnγ* ^−/−^ *pfn* ^+/+^) or Pfn-deficient (*ifnγ* ^+/+^ *pfn* ^−/−^) mice.

2 ECG parameters were evaluated using the following standard criteria: (i) heart rate (monitored by beats per minute (bpm), and (ii) the variation of the P wave and PR, QRS and corrected QT intervals (QTc), all measured in milliseconds (ms). ART, arrhythmia; AVB1, first-degree atrioventricular block; AVB2, second-degree atrioventricular block.

3 This table represented accumulated data from three independent experiments, with 3 to 5 mice/group in each experiment.

4 Significant differences:

*: , *p*<0.05 - between the values for *cd8* ^−/−^ noninfected and *T. cruzi*-infected mice;

# , *p*<0.05 - between the values for *cd8* ^−/−^ non-reconstituted and *cd8* ^−/−^ reconstituted and infected with *T. cruzi*.

Interestingly, IHS analysis showed that the reconstitution of *cd8* ^−/−^ mice with CD8^+^ cells from *ifnγ* ^+/+^ *pfn* ^−/−^ donors led to a significant accumulation of IFNγ^+^ cells in the cardiac tissue (**Figure 7F**); however, a low number of IFNγ^+^ cells was also detected in the cardiac tissue of *cd8* ^−/−^ mice that were non-reconstituted or reconstituted with CD8^+^ T-cells from *ifnγ* ^−/−^ *pfn* ^+/+^donors, supporting the existence of non-CD8 IFNγ producers in the *cd8* ^−/−^ mice (probably NK or CD4^+^ cells). Importantly, in the *cd8* ^−/−^ mice that were reconstituted with CD8^+^ T-cells from *ifnγ* ^−/−^ *pfn* ^+/+^ donors, Pfn^+^ cells accumulated in the cardiac tissue (**Figure 7G**). No Pfn^+^ cells were detected in the cardiac tissue of non-reconstituted or *cd8* ^−/−^ mice that were reconstituted with CD8^+^ cells from *ifnγ* ^+/+^ *pfn* ^−/−^ donors (**Figure 7G**), supporting that all Pfn^+^ cells detected in the cardiac tissue were CD8^+^ T-cells. Because reductions in cardiomyocyte lesion and electrical alterations were observed, the data support the finding that CD8^+^ IFNγ producers colonizing the cardiac tissue play a beneficial role by decreasing heart injury during *T. cruzi* infection. Conversely, the aggravation of cardiomyocyte lesion revealed by increased CK-MB activity levels in the serum detected in *cd8* ^−/−^ mice that had been reconstituted with CD8^+^ T-cells from *ifnγ* ^−/−^ *pfn* ^+/+^ donors (**Figure 7E**) was associated with an accumulation of Pfn^+^ cells in the cardiac tissue (**Figure 7G**), reinforcing that CD8^+^Pfn^+^ cells play a detrimental role in heart injury during *T. cruzi* infection.

## Discussion

In Chagas disease, CD8^+^ T-cells are crucial for *T. cruzi* dissemination control during the acute infection phase. In the chronic infection the predominance of CD8^+^ T-cells among the cells infiltrating the cardiac tissue raised the suspicion that these cells are involved in heart injury. In this study, we adopted a murine model of *T. cruzi*-elicited chronic cardiomyopathy to examine the participation of CD8^+^ T-cells that express IFNγ and Pfn in parasite control and heart injury. There was no association of the intensity of heart parasitism and CD8-enriched myocarditis with cardiomyocyte lesion and electrical abnormalities during the chronic infection. Furthermore, the expansion and contraction of anti-VNHRFTLV CD8^+^ T-cell effector activities (IFNγ-producers and CTL) in the periphery (spleen and blood) were associated with the parasite load but not with heart injury. The presence, however, of anti-VNHRFTLV CD8^+^ effector T-cells in the cardiac tissue paralleled the tissue damage and electrical abnormalities. The accumulation of IFNγ^+^ cells preceded the entry of Pfn^+^ cells into the cardiac tissue during acute infection. Interestingly, in the chronic infection, a decrease in IFNγ^+^ and a relative enrichment in Pfn^+^ cells invading the cardiac tissue paralleled the worsening of heart injury. In fact, there was prevalent accumulation of CD8^+^Pfn^+^ cells in the cardiac tissue in *T. cruzi* infection. Moreover, there is a differential accumulation of CD8^+^ H-2K^b^/VNHRFTLV^+^ Pfn^+^ T-cells in the cardiac tissue during the chronic infection. Altogether, these findings led to the idea that CD8^+^Pfn^+^ and CD8^+^IFNγ^+^ exhibit a distinct migratory behavior. Corroborating this theory, CD8^+^ cells from *T. cruzi*-infected *ifnγ* ^−/−^ *pfn* ^+/+^ donors (Pfn^+^) migrate towards the cardiac tissue more so than do CD8^+^ cells from *ifnγ* ^+/+^ *pfn* ^−/−^ donors (IFNγ^+^). Moreover, we demonstrate that the colonization of cardiac tissue of *cd8* ^−/−^ mice by CD8^+^Pfn^+^ cells aggravated cardiomyocyte injury, whereas CD8^+^IFNγ^+^ cells ameliorated cardiomyocyte injury and electrical abnormalities, thus supporting a differential role for CD8^+^Pfn^+^ and CD8^+^IFNγ^+^ T-cells in *T. cruzi*-elicited cardiomyopathy.

Following infection of C57BL/6 mice with the Colombian strain of *T. cruzi*, parasite load was controlled and most of the animals survived the acute phase of infection and developed a chronic infection. In these animals, myocardial cell damage and electrical abnormalities, such as arrhythmias and prolonged QTc interval, are similar to those described in CCC patients [2], [24] and other murine models of *T. cruzi* infection [12], [20]. Moreover, in *T. cruzi*-infected C57BL/6 mice, chronic CD8-enriched myocarditis was associated with cardiomegaly. Taken together, these data validate this model for study of the immunopathology of Chagas' heart disease.

Our first goal was to determine whether the development of chronic cardiomyopathy, characterized by the presence of cardiomyocyte lesion and electrical abnormalities, was associated with the intensity of the parasite load in cardiac tissue and/or CD8-enriched myocarditis. During the acute infection phase of C57BL/6 with the Colombian strain of *T. cruzi*, cardiomyocyte lesion and electrical alterations were related to heart parasitism and inflammation. This finding partially corroborated previous data using a model of acute infection. Briefly, they infected Swiss mice with the Y strain of *T. cruzi* and observed a positive correlation between the intensity of heart inflammation, but not heart parasitism, and CK-MB activity levels in plasma [16]. Therefore, in C57BL/6 mice acutely infected with the Colombian strain of *T. cruzi*, heart injury may be the consequence of the direct destruction of cardiomyocytes by intense parasitism and/or anti-*T. cruzi* effector immunity. However, as infection progresses, heart parasitism and CD8-enriched myocarditis decreases, coinciding with the establishment of anti-*T. cruzi* specific CD8^+^ T-cell effector activities (inflammatory and cytotoxic) and parasitemia control, whereas myocardial cell damage and electrical abnormalities are aggravated. These data confirm the lack of an association between the intensity of heart parasitism and myocarditis with the evolution of heart injury in the chronic phase of *T. cruzi* infection, as previously seen in cardiopatic chagasic patients [2], [5], [25] and C3H/He mice infected with the Colombian strain of *T. cruzi* [12]. Therefore, our data reinforce the concept that long-term heart inflammation, rather than its intensity, is crucial for the generation of *T. cruzi*-elicited heart injury.

In spite of their importance for host resistance in *T. cruzi* infection [4], CD8^+^ T-cells gained particular attention as the major component of myocarditis in acute [26] and chronic [6] experimental infection, and in chronic chagasic patients [5], [13]. Therefore, we compared the kinetics of the generation of inflammatory (IFNγ producers) and cytotoxic CD8^+^ T-cell effectors specific for the immunodominant VNHRFTLV ASP2 epitope, which is linked to protective immunity against *T. cruzi* [27], [28] and is a prototype for general CD8^+^ T-cell activation, with the kinetics of the appearance of cardiomyocyte lesions and electrical abnormalities. The H-2K^b^-restricted anti-VNHRFTLV cytotoxic and IFNγ-secreting CD8^+^ T-cells were first detected before the peak of parasitemia at 15 dpi and reached a maximum level at the peak of parasitemia and heart parasitism (42–45 dpi). This finding contrasts with previous data showing that the peak of anti-VNHRFTLV cytotoxic and IFNγ-secreting CD8^+^ T-cells occurred after the peak of parasitemia (between 14 and 24 dpi in the studied strains), implying that there is a requirement for rounds of parasite multiplication to trigger CD8^+^ T-cell effector activities [29]. In light of these data, it appears that during *T. cruzi* infection, specific CD8^+^ T-cell effectors (IFNγ producers and cytolytic effectors) require between 15 and 20 dpi to proliferate and differentiate independent of the mouse lineage, parasite strain (virulence and inoculum size) and parasite load requirements. As expected, after parasite control (60 dpi) CD8^+^ T-cell effectors (IFNγ producers and cytolytic effectors) decreased but persisted at detectable levels up to 120 dpi. Importantly, in the periphery the maximum anti-VNHRFTLV ASP2 epitope CD8^+^ T-cell effector cytotoxic and inflammatory activities was achieved at the parasitemia peak (42–45 dpi) and was probably stimulated by the proportional release of parasite antigens. Importantly, it was not kinetically related to the more severe heart injury that was observed at a later time (90 dpi). Therefore, protective CD8^+^ T-cell effectors controlling *T. cruzi* growth in the periphery and detrimental myocarditis appear to be dissociated facets of the host immune response that is triggered by the invading organism [12], [23]. If so, one may be able to stimulate anti-parasite protective immunity and selectively downregulate the detrimental inflammation colonizing the cardiac tissue. Therefore, an understanding of the functional role played by the cells invading the cardiac tissue is required. Our next goal was to establish the kinetics of the heart colonization by inflammatory cells during *T. cruzi* infection to determine the association with heart injury. CD8-enriched myocarditis was established during acute infection and although inflammation decreased as a whole, CD8^+^ T-cells persisted as the predominant cell population invading the cardiac tissue during the chronic phase of infection, corroborating findings observed in patients [5], [13] and C3H/He mice infected with the Colombian *T. cruzi* strain [6]. Previous data demonstrated that IFNγ is crucial for regulation of the entry of anti-parasite protective cells into the cardiac tissue [11], and a Pfn deficiency results in less severe cardiomyocyte lesion and electrical abnormalities during chronic *T. cruzi* infection [7]. Therefore, we investigated whether IFNγ^+^ and Pfn^+^ cells colonize the cardiac tissue simultaneously or in a dissociated manner during *T. cruzi* infection, and examined the possible implications of this scenario in chronic cardiomyopathy. The entry of IFNγ^+^ cells into the cardiac tissue preceded the influx of Pfn^+^ cells, suggesting that inflammatory and cytotoxic effector activities are performed by distinct cell populations during a *T. cruzi* infection. Indeed, most of the CD8^+^ T-cells that were detected in the peripheral blood of acute and chronically *T. cruzi*-infected mice were segregated into CD8^+^IFNγ^+^ and CD8^+^Pfn^+^ cell populations. Importantly, these *ex vivo* stained cells reflect the status of *in vivo* activated cells that are present in peripheral blood and migrate to tissues that are targeted by *T. cruzi*, e.g., the cardiac tissue. Furthermore, this segregation is a long-term feature as opposed to a transient characteristic of distinct inflammatory (IFNγ producers) and cytotoxic (Pfn-expressing) CD8^+^ T-cell effectors in acute and chronic *T. cruzi* infection. In fact, in the cardiac tissue the accumulation of segregated CD8^+^Pfn^+^ T-cells observed in the acute infection persisted in the chronic phase of infection. Moreover, the CD8^+^IFNγ^+^ T-cells detected in the cardiac tissue are IFNγ^dull^. Thus, this pale expression of IFNγ by the inflammatory cells invading the cardiac tissue, that confirmed previous data [30], may be related to continuous *in situ* antigenic stimulation.

The possibility of coexpression of IFNγ and Pfn by a single CD8^+^ T-cell under antigen re-stimulation conditions cannot be ruled out. Indeed, there was a low but consistent increased frequency of CD8^+^ T-cells in the peripheral blood *T. cruzi*-infected mice (from 0.01–0.05% in noninfected to 0.19–0.34% in chronically infected mice). Further, anti-*T. cruzi* CD8^+^IFNγ^+^Pfn^+^ T-cells were retained in the spleen during chronic infection. In this context, a recent report showed that following the *in vitro* restimulation with human immunodeficiency virus antigens, peripheral blood CD8^+^ T-cells from elite controllers presented a higher frequency of IFNγ^+^Pfn^+^ responders than CD8^+^ T-cells from chronic progressors [31]. Therefore, it remains to be determined whether the segregation of effector activities in CD8^+^IFNγ^+^ and CD8^+^Pfn^+^ T-cells is a *T. cruzi*-driven feature that contributes to parasite escape and persistence in chronic infection. Furthermore, if this is the case, it is reasonable to suppose that the stimulation of anti-*T. cruzi* immunity during chronic infection, for example, through immunotherapeutic vaccination, would upregulate multifunctional CD8^+^IFNγ^+^Pfn^+^ T-cells.

A predominance of CD8^+^IFNγ^+^ cells compared with CD8^+^Pfn^+^ T-cells was consistently detected in the peripheral blood of C57BL/6 mice during the acute and chronic phases of *T. cruzi* infection. Independent of the lower frequency of CD8^+^Pfn^+^ cells available in the blood, we noticed a gradual relative accumulation of Pfn^+^ cells in the cardiac tissue as the *T. cruzi* infection evolved into the chronic phase. Therefore, we were interested in determining whether these CD8^+^ T-cells expressing IFNγ^+^ and Pfn^+^ in a segregated form represent a distinct status of molecules involved in the process of cell migration. Indeed, CD8^+^Pfn^+^ T-lymphocytes have a higher frequency of CCR5^+^LFA-1^+^ cells compared with CD8^+^IFNγ^+^ T-cells. Interestingly, there was a decrease in the proportion of CD8^+^Pfn^+^ cells and an increase in the frequency of CD8^+^IFNγ^+^ cells in the peripheral blood during the chronic infection. Importantly, there was accumulation of CD8^+^Pfn^+^ cells in relation to CD8^+^IFNγ^+^ cells in the cardiac tissue independently of the prevalence of CD8^+^IFNγ^+^ cells in the peripheral blood of chronically infected mice. A high frequency of CD8^+^IFNγ^+^ cells in the peripheral blood of patients with severe CCC was interpreted as participation of this population of cells in immunopathogenesis [32]. However, we believe that this concept should be re-interpreted because the evidence suggests that the findings in the peripheral blood do not reflect the scenario of the cellular environment of the cardiac tissue.

The intensity of acute and chronic *T. cruzi*-elicited myocarditis is related to the concentrations of the CC-chemokines CCL3/MIP-1α and CCL5/RANTES, but not to the concentrations of IFNγ and TNF in the cardiac tissue [12]. Furthermore, 100% of the CD8^+^ cells that colonize the heart tissue express the CC-chemokine receptor CCR5 [23], [33] and the cell adhesion molecule LFA-1 [6], suggesting that CCR5^+^LFA-1^+^ cells colonized the cardiac tissue during *T. cruzi* infection and retain this activation phenotype. Most of the cardiomyocytes express the LFA-1 ligand ICAM-1 [34], [35]. Moreover, besides being key molecules in cell migration, LFA-1 and CCR5 play roles in immunological synapses and cell activation [36], [37]. Therefore, both CCR5 and LFA-1 expressed by CD8^+^ effector T-cells (either IFNγ^+^ or Pfn^+^) may play roles in parasite control or cytotoxic activity in the cardiac tissue. ICAM-1-deficient mice exhibit poor migration of CD4^+^ and CD8^+^ T-cells towards the cardiac tissue, where the parasite is less controlled [11]. In addition, *T. cruzi*-infected CCR5-deficient mice experience dramatically inhibited migration of T-cells to the cardiac tissue and are more susceptible to infection, demonstrating that CCR5 and its ligands play a central role in the control of T-cell influx towards the cardiac tissue in *T. cruzi*-infected mice [33], [38]. However, the partial blockage of the CC-chemokine receptors CCR1/CCR5 using Met-RANTES during *T. cruzi* infection was beneficial as it did not interfere with the control of heart parasitism but significantly reduced the numbers of CD4^+^, CD8^+^ and CCR5^+^ lymphocytes in the cardiac tissue during acute infection [23]. During chronic infection, Met-RANTES inhibited connexin 43 loss and decreased cardiomyocyte lesion [12]. These data suggest that not all CCR5^+^ cells are crucial for parasite control, and at least some of the cells play a pivotal role in the pathogenesis of *T. cruzi*-elicited cardiomyopathy. In this context, our data indicate that because a larger proportion of CD8^+^Pfn^+^ cells than CD8^+^IFNγ^+^ cells were CCR5^+^LFA-1^+^, these CD8^+^Pfn^+^ cells may exhibit a differential migratory behavior that favors their accumulation in the cardiac tissue, contributing to explain the observed accumulation of CD8^+^Pfn^+^ cells in the cardiac tissue in chronically *T. cruzi*-infected C57BL/6 mice. The adoptive cell transfer of CD8^+^ T-cells from *ifnγ* ^−/−^ *pfn* ^+/+^ and *ifnγ* ^+/+^ *pfn* ^−/−^ infected donors to C57BL/6 and *cd8* ^−/−^ infected recipient mice circumstantially support this idea because CD8^+^ T-cells from *ifnγ* ^−/−^ *pfn* ^+/+^ infected donors (expressing Pfn) accumulated in the cardiac tissue to a greater extent than CD8^+^ T-cells that had been isolated from *ifnγ* ^+/+^ *pfn* ^−/−^ infected donors (expressing IFNγ). Therefore, this differential migratory behavior of CD8^+^ cells that express Pfn, but do not produce IFNγ, might explain the gradual relative accumulation of Pfn^+^ cells in the cardiac tissue during *T. cruzi* infection. This was confirmed by the flow cytometry study showing the accumulation of CD8^+^Pfn^+^ T-cells in relation to CD8^+^IFNγ^+^ cells in the cardiac tissue. Furthermore, in the cell transfer experiments, we noticed that independent of the heart parasitism that remained equal in all experimental groups, the differential entry of CD8^+^ T-cells into the cardiac tissue was sufficient to produce distinct cardiomyocyte lesion profiles at 10 days after cell transfer. Interestingly, in both C57BL/6 and *cd8* ^−/−^infected recipient mice, CD8^+^ T-cells from *ifnγ* ^−/−^ *pfn* ^+/+^ infected donors (produce Pfn) were implicated in myocardial cell damage, whereas CD8^+^ T-cells from *ifnγ* ^+/+^ *pfn* ^−/−^ infected donors (produce IFNγ) contributed to the amelioration of cardiomyocyte lesion. Interestingly, as heart parasitism was similar in these groups, the beneficial role of the CD8^+^ T-cells originating from *ifnγ* ^+/+^ *pfn* ^−/−^ infected donors was not due to the parasite control in this case. Therefore, these cells might favor cardiomyocyte integrity through other mechanisms that remain to be explored. A recent study supports that at 30 dpi a proportion of infiltrating cardiac cells coexpress IL-10 and IFNγ and may play a beneficial role in *T. cruzi*-elicited myocarditis [22]. In our study, although we were able to detect CD8^+^ cells expressing IL-10 in the cardiac tissue at 45 and 120 dpi of C57BL/6 mice with the Colombian *T. cruzi* strain, only a small proportion of the IL-10^+^ cells coexpress IFNγ^dull^, while in the peripheral blood the majority of IL-10^+^ cells coexpress IFNγ^bright^. Thus, the putative participation of these cells in heart injury in *T. cruzi* infection requires further exploration.

Finally, to approach the differential contribution of IFNγ^+^ and Pfn^+^ cells to heart injury, we reconstituted the CD8^+^ cell compartment of *cd8* ^−/−^ mice with CD8^+^ T-cells from *ifnγ* ^−/−^ *pfn* ^+/+^ and *ifnγ* ^+/+^ *pfn* ^−/−^ donors prior to *T. cruzi* infection. During the acute infection, the reconstitution of *cd8* ^−/−^ mice with CD8^+^ T-cells from *ifnγ* ^−/−^ *pfn* ^+/+^ donors (deficient in IFNγ but able to express Pfn) significantly favored the control of the parasite when compared with non-reconstituted mice, corroborating the reports that suggest the importance of Pfn in the protective immunity that controls *T. cruzi* during the acute phase of infection [7], [28]. Importantly, when naïve *cd8* ^−/−^ mice were reconstituted with CD8^+^ cells from *ifnγ* ^+/+^ *pfn* ^−/−^ naïve donors (deficient in Pfn but able to express IFNγ), after infection these animals exhibited less severe myocardial cell lesion and a more preserved electrical conduction, with a low incidence of AVB2 and a more normal heart rate. The reconstitution of naïve *cd8* ^−/−^ mice with CD8^+^ cells from *ifnγ* ^−/−^ *pfn* ^+/+^ mice (deficient in IFNγ but able to express Pfn) prior to infection had no impact on electrical conduction, which were similar to those found in NR *cd8* ^−/−^ mice, but aggravated cardiomyocyte injury. Thus, our data support that CD8^+^Pfn^neg^IFNγ^+^ cells act beneficially towards cardiomyocytes, whereas CD8^+^Pfn^+^IFNγ^neg^ cells have a detrimental effect on cardiomyocyte lesion in reconstituted *T. cruzi*-infected mice. Importantly, our data showing the prevalence of CD8^+^Pfn^+^IFNγ^neg^ cells in the cardiac tissue in chronic *T. cruzi* infection of C57BL/6 mice circumstantially supports a detrimental role for these cells in heart injury.

Emerging evidence supports that cytotoxic (Pfn^+^, granzyme^+^) and pro-inflammatory (IFNγ^+^) CD8^+^ T-cell effectors may differentially determine the outcome of infectious processes [15], [30]. In Chagas disease, the contribution of Pfn^+^ and IFNγ^+^ cells to heart injury has not been determined. IFNγ-producing CD8^+^ cells have been linked with protective immunity against *T. cruzi* reinfection in endemic areas [8] and with a benign clinical outcome in patients with the indeterminate and less severe form of CCC [9]. Conversely, CD8^+^IFNγ^+^ cells have been associated with the severity of CCC [32], [39]. IFNγ-deficient mice are less resistant to *T. cruzi* infection than their wild-type counterparts [40], [41], reinforcing the concept that IFNγ participates in parasite control. *In vitro* experiments showed that IFNγ acts directly on *T. cruzi*-infected cardiomyocytes, inducing nitric oxide production and low trypanocidal activity, which was enhanced by addition of IL-1β, TNF or CC-chemokines [42]. On the other hand, the presence of granzyme A^+^ cytotoxic CD8^+^ cells in the cardiac tissue is strongly associated with CCC severity [13]. In this context, recent findings support the idea that although Pfn takes part in *T. cruzi* growth control, this member of the lytic machinery is not crucial because *ifnγ* ^+/+^ *pfn* ^−/−^ mice survived the acute infection and developed a less severe chronic cardiomyopathy in an IFNγ-enriched milieu [7]. However, the direct protective effect on parasite control and the beneficial or detrimental actions of CD8^+^IFNγ^+^ and CD8^+^Pfn^+^ cells on heart injury during *T. cruzi* infection have not been explored. In this study, we present circumstantial evidence indicating that the enrichment in H-2K^b^/VNHRFTLV^+^ Pfn^+^ cells among heart infiltrating inflammatory cells paralleled the chronic myocardial cell injury and electrical abnormalities in chronically *T. cruzi*-infected C57BL/6 mice, while H-2K^b^/VNHRFTLV^+^ IFNγ^+^ cells were retained in the spleen. Hence, it is conceivable to propose that in this unbalanced compartmentalization of the *T. cruzi* specific IFNγ^+^ cells may relay the perpetuation of Chagas'heart disease, deserving further investigation.

In summary, we demonstrate that during *T. cruzi* infection, major inflammatory and cytotoxic effector activities are performed mainly by segregated CD8^+^IFNγ^+^ and CD8^+^Pfn^+^ T-cell populations. In addition, CD8^+^Pfn^+^ cells exhibit a more frequent CCR5^+^LFA-1^+^ migratory profile that might favor the entrance and accumulation of CD8^+^Pfn^+^ cells in the cardiac tissue during infection. Moreover, our data support the concept that CD8^+^Pfn^+^ T-cells are involved in cardiomyocyte injury during *T. cruzi* infection, whereas CD8^+^IFNγ^+^ cells play a beneficial role in cardiomyocyte damage. Recent *in vitro* findings showed that anti-TNF therapy decreases Pfn expression in CD8^+^ T-cells [43]. Therefore, it is reasonable to hypothesize that *in vivo* therapeutic interventions could selectively interfere with distinct CD8^+^ T-cell effectors, which could hamper the massive entry of deleterious anti-*T. cruzi* Pfn^+^ expressing cells in the cardiac tissue but improve anti-*T. cruzi* protective immunity and play a benefic role against cardiomyocyte injury, thus opening a new avenue to be explored in Chagas' heart disease therapy. Lastly, we believe that distinguishing the CD8^+^ T-cells effectors that contribute to the occurrence of chronic heart lesions is a crucial goal, which will be of inestimable prognostic value for CCC patients.

## Materials and Methods

### Ethics statement

This study was carried out in strict accordance with the recommendations in the Guide for the Care and Use of Laboratory Animals of the Brazilian National Council of Animal Experimentation (http://www.cobea.org.br/) and the Federal Law 11.794 (October 8, 2008). The Institutional Committee for Animal Ethics of Fiocruz (CEUA/Fiocruz, License 004/09) and the Biosafety National Committee (CQB/CTNBio, License 105/99) approved all the procedures used in this study.

### Mice

Female C57BL/6 (H-2^b^), Pfn-deficient (*ifnγ* ^+/+^ *pfn* ^−/−^, B6.129-pfn-^tm1Clrk−/−^), IFNγ-deficient (*ifnγ* ^−/−^ *pfn* ^+/+^) and CD8-deficient (*cd8* ^−/−^) mice, all in the C57BL/6 (B6) genetic background and aged between five and seven weeks, were obtained from the Oswaldo Cruz Foundation animal facilities (CECAL, Rio de Janeiro, Brazil) and were maintained under specific pathogen free conditions. In all sets of experiments three to five sex- and age-matched noninfected controls were analyzed per time point in parallel to five to ten infected mice, according to the experimental protocol.

### Experimental infection

C57BL/6, *ifnγ* ^+/+^ *pfn* ^−/−^ and *ifnγ* ^−/−^ *pfn* ^+/+^ mice were infected with the Colombian *T. cruzi* strain by the intraperitoneal injection of 100 blood trypomastigotes (bt). Parasitemia was estimated in 5 µL of tail vein blood. After the peak of parasitemia, the detection of rare circulating trypomastigotes marked the onset of the chronic phase of infection, as previously described [6].

### Reagents and antibodies

For functional assays, the H-2K^b^-restricted VNHRFTLV peptide from the amastigote surface protein 2 (ASP2) [16] was synthesized by GenScript USA Inc. (Piscataway, NJ, USA). For flow cytometry analysis, we used the biotinylated major histocompatibility complex class I (MHC I) multimer H-2K^b^/VNHRFTLV produced by Proimmune (Oxford, UK), previously used to access the specific anti-*T. cruzi* immune response [21], according to manufacturer's instructions. For use in immunohistochemistry staining (IHS), polyclonal antibody that recognizes *T. cruzi* antigens and anti-mouse CD8a (53-6.7) and anti-mouse CD4 (GK1.5) supernatants were produced in our laboratory (LBI/IOC-Fiocruz, Brazil). The monoclonal antibodies anti-mouse Pfn (CB5.4, Alexis Biochemicals, San Diego, CA, USA) and anti-IFNγ (R4-6A2, BD PharMingen, San Diego, CA, USA) were also used in IHS. The biotinylated anti-rat immunoglobulin was purchased from DAKO (Glostrup, Denmark), and the biotinylated anti-rabbit immunoglobulin and peroxidase-streptavidin complex were acquired from Amersham (Buckinghamshire, England). Appropriate controls were prepared by replacing the primary antibodies with purified rat immunoglobulin or normal rabbit serum. For *in vivo* cytotoxicity assays and immunofluorescence staining of the cells to be used in the adoptive cell transfer assays, we used a cell trace TM CFSE cell proliferation kit (C34554) for flow cytometry (Invitrogen, Carlsbad, CA, USA). For CD8^+^ T-cell enrichment by positive selection, we used mouse CD8 Dynabeads (LyT-2) and magnetic separation Dynal MPC (Invitrogen, Carlsbad, CA, USA). For flow cytometry studies, FITC-, PE-conjugated isotype controls Rat IgG, PE- and FITC-conjugated anti-T-cell receptor αβ (clone H57-597), PE- and FITC-conjugated anti-CD4 (L3T4, clone GK1.5), PE- and APC-conjugated anti-CD8α (Ly-2, clone 53-6.7), FITC-conjugated anti-NK 1.1 (NKR-P1B and NKR-P1C, clone PK136), PECy-7-conjugated anti-IFNγ (XMG1.2), PECy-7-conjugated anti-TNF (MP6-XT22), FITC-conjugated anti- Pfn (11B11), PE-cy7-conjugated anti-LFA-1 (2D7) and PE-conjugated anti-CCR5 (clone C34-3448) were purchased from BD PharMingen (San Diego, CA, USA), FITC-conjugated anti-LFA-1 (CD11a/CD18b, clone M17/4) and PE-conjugated anti-IL-10 (clone LRM9104) were obtained from CALTAG (Burlingame, CA,USA), PE-conjugated anti-CD107a (eBIO1D4B) was purchased from eBioscience (San Diego, USA). Appropriate controls were prepared by replacing the primary antibodies with their respective isotypes obtained from BD PharMingen (San Diego, CA, USA) or from SouthernBiotech (Alabama, USA). Endotoxin-free purified anti-CD3 (clone 145 – 2C11) and anti-CD28 (clone 37.51) were purchased from SouthernBiotech (Alabama, USA).

### Immunohistochemistry (IHS)

Groups of five to seven infected and three to five noninfected age-matched control mice were sacrificed under anesthesia at various time points after infection. The hearts of the mice were removed, embedded in tissue-freezing medium (Tissue-Tek, Miles Laboratories, Elkhart, IN, USA) and stored in liquid nitrogen for analysis by immunohistochemistry. Serial cryostat sections, 3 µm-thick, were fixed in cold acetone and subjected to indirect immunoperoxidase staining, as previously described [6].

For negative controls, heart tissue sections from experimentally infected mice were subjected to similar treatments as the control samples except primary antibodies were omitted from the reactions. Sections of the spleen were used as positive controls for lymphocyte staining. For each tissue section, the number of IFNγ^+^ inflammatory cells and Pfn^+^ cells were counted in 50 microscopic fields (400× magnification). Perforin staining was performed in accordance with the manufacturer's instructions. The presence of *T. cruzi* in cardiac tissue sections was evaluated using a digital morphometric apparatus. The images were analyzed using the AnaliSYS Program and the areas containing parasite molecules were identified as amastigote nests. For each heart sample, three separate tissue sections and 50 fields per section were analyzed. The number of amastigote nests was determined in 100 microscopic fields (magnification 400×) per tissue section.

### Cell preparation and intracellular cytokine staining by flow cytometry analysis (FACS)

The mice were sacrificed by blood draining under anesthesia and the harvested spleens were teased into single cell suspensions as previously described [6]. For *ex vivo* analysis, peripheral blood cells and splenocytes were incubated with 5 µg/mL brefeldin A (Sigma, St. Louis, MO, USA) for 4 hours at 37°C. The cells were collected, washed, resuspended in PBS containing 2% fetal calf serum and labeled as previously described [6]. To prepare mononuclear cell suspensions from cardiac tissue, 5–10 hearts were washed to remove blood clots, minced with scissors into 1–2 mm fragments and subjected to enzymatic digestion using a solution containing 0.015% trypsin (T4799; Sigma, St. Louis, MO, USA) and collagenase A (103586; Boehringer, Mannheim, Germany), as previously described [6]. In all experiments, one-color labeled samples were prepared for establishment of the compensation values. The controls for specific labeling were prepared using isotype-matched antibodies. The samples were acquired using a BD FACScalibur (BD PharMingen, San Diego, CA, USA) and the Beckman Coulter CyAn 7 Color flow cytometer (Fullerton, CA, USA). The mononuclear cells were gated and a narrow forward angle light scatter parameter was used to exclude dead cells from the analysis. At least 100,000 cells were acquired inside this gate. The fluorescence gates were cut in accordance with the labeling controls, respecting the curve inflexions. The flow cytometric analysis was carried out using the Summit v.4.3 Build 2445 program (Dako, Glostrup, Denmark).

### IFN-γ enzyme-linked immunospot (ELISpot) assay

The ELISpot assay for the enumeration of IFNγ-producing cells was performed as previously described [17]. The assays were performed in triplicate. The plates were coated with anti-mouse IFNγ (clone R4-6A2; BD PharMingen, San Diego, CA, USA) antibody diluted in PBS (5 µg/mL). Further, the antigen presenting cells were primed with total *T. cruzi* antigens (10 µg/mL) for 30 minutes at 37°C. Concanavalin A (ConA, 5 µg/mL) was used as a mitogenic stimulant. After incubation, the freshly isolated splenocytes were seeded at a suspension of 5×10^5^ cells/well and were incubated with the H-2K^b^-restricted VNHRFTLV peptide from ASP2 for 20 hours at 37°C and 5% CO_2_. A biotin-conjugated anti-mouse IFNγ antibody (clone XMG1.2; BD PharMingen, San Diego, CA, USA) was used to detect the captured cytokines. The spots were revealed by respective incubation of the samples with a solution of alkaline phosphatase-labeled streptavidin (BD PharMingen, San Diego, CA, USA) and a solution of nitro blue tetrazolium (NBT; Sigma, St. Louis, MO, USA) and 5-Bromo-4-chloro-3-indolyl phosphate (BCIP; Sigma, St. Louis, MO, USA) in Tris buffer (0.9% NaCl, 1% MgCl_2_, 1.2% Tris in H_2_O). The mean number of spots in triplicate wells was determined for each experimental condition and the number of specific IFNγ-secreting T-cells was calculated by estimating the stimulated spot count / 10^6^ cells using a CTL OHImmunoSpot A3 Analyzer (Cleveland, OH, USA).

### *In vivo* cytotoxicity assay

For the *in vivo* cytotoxicity assays, splenocytes collected from naïve C57BL/6 mice were treated with ACK buffer (Sigma, St. Louis, MO, USA) to lyse the red blood cells. The cells were divided into two populations and were labeled with the fluorogenic dye carboxyfluorescein diacetate succinimidyl diester (CFSE; Molecular Probes, Eugene, OR, USA) at a final concentration of 5 µM (CFSE^high^) or 0.5 µM (CFSE^low^). CFSE^high^ cells were coated with 2.5 µM of the VNHRFTLV ASP2 peptide for 40 minutes at 37°C. CFSE^low^ cells remained uncoated. Subsequently, CFSE^high^ cells were washed and mixed with equal numbers of CFSE^low^ cells before intravenous injection (1–2×10^7^ cells per mouse) into *T. cruzi*-infected C57BL/6 recipients that were sedated with diazepam (20 mg/Kg). Spleen cells from the recipient mice were collected at 20 hours after adoptive cell transfer, as indicated in the figure legends, fixed with 1.0% paraformaldehyde and analyzed using the Summit v.4.3 Build 2445 program (Dako, Glostrup, Denmark). The percentage of specific lysis was determined using the following formula:
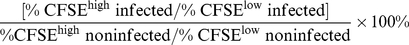



### CD8 T-cell enrichment and *in vivo* leukocyte migration assay

The spleens were removed from *ifnγ* ^+/+^ *pfn* ^−/−^ or *ifnγ* ^−/−^ *pfn* ^+/+^ donor mice at 20 days post-infection (dpi). Single-cell suspensions of splenocytes were prepared and the red blood cells were lysed using ACK buffer (Sigma, St. Louis, MO, USA). The splenocyte suspensions were enriched for CD8^+^ cells by positive selection (Dynabeads mouse CD8, Invitrogen Dynal AS, Oslo, Norway). Briefly, 8×10^7^ splenic leukocytes / mL were labeled with a CD8^+^ enrichment antibody mixture conjugated to magnetic beads and separated with the Dynal MPC apparatus (Invitrogen Dynal AS, Oslo, Norway) according to manufacturer's recommendations. CD8-enriched cells were separated from the beads by incubating the bead-coated cells overnight at 37°C. After incubation, the tubes containing the cell suspensions were placed in the Dynal MPC magnetic separator (Invitrogen Dynal AS, Oslo, Norway) for 2 minutes and the bead-free cells were removed from the supernatant. The CD8-enriched cells were washed three times in sterile saline and used for adoptive transfer. The purity and percentage of CD8^+^ T-cells, and the expression of IFNγ and Pfn, were determined by flow cytometry. For the adoptive cell transfer experiments, only cell suspensions that contained a CD8-enrichment of ≥98% were used. The CD8^+^ T-cells were labeled with 10 µM CFSE as described above. The cell suspensions (5×10^6^ cells per mouse) were intravenously injected into C57BL/6 and *cd8* ^−/−^ recipient mice at 20 dpi, that had been previously sedated with diazepam (20 mg/Kg). As a control, C57BL/6 and *cd8* ^−/−^ mice at 20 dpi received intravenous injections of 100 µL of sterile saline. The mice were euthanized at 3, 7 and 10 days after the adoptive cell transfer (23, 27 and 30 dpi, respectively), the hearts were removed, and were embedded in tissue-freezing medium (Tissue Tek, Miles Laboratories, Elkhart, IN, USA). Serial 3 µm-thick sections were prepared, and were fixed in cold acetone. The nuclear DNA was stained with DAPI (1 mg/mL in PBS) in the presence of DABCO anti-fading agent (Sigma, St. Louis, MO, USA). Using a confocal microscope (LSM 410; Carl Zeiss, Stuttgart, Germany), the number of CFSE^+^ cells in 100 microscopic fields (magnification 400×) was determined and the intensity of fluorescence determined using the program LSM Image Browser (Carl Zeiss, Stuttgart, Germany).

### CFSE-based lymphoproliferative response

The lymphoproliferative response was assessed as previously described [44]. Briefly, the spleens were removed from noninfected or *T. cruzi*-infected (20 dpi) C57BL/6, *ifnγ* ^+/+^ *pfn* ^−/−^ or *ifnγ* ^−/−^ *pfn* ^+/+^ mice, single-cell suspensions of splenocytes were prepared, the red blood cells were lysed using ACK buffer (Sigma, St. Louis, MO, USA) and the monononuclear cells labeled with CFSE at a final concentration of 7 µM (CFSE^high^) or 0.5 µM (CFSE^low^). The cells were incubated in RPMI medium supplemented with 10% SBF in the presence of anti-CD3 and anti-CD28 (3 µg/mL) or 2.5 µM of the VNHRFTLV ASP2 peptide during 72 hours at 37°C and 5% CO_2_. The CFSE^high^ cells were washed and fixed with 1.0% paraformaldehyde. The CFSE^low^ cells were washed and labeled with APC-conjugated anti-CD8 antibody as described above, washed and fixed with 1.0% paraformaldehyde. All the samples were acquired using a Beckman Coulter CyAn 7 Color flow cytometer (Fullerton, CA, USA) and analyzed using the Summit v.4.3 Build 2445 program (Dako, Glostrup, Denmark).

### CD8^+^ T-cell compartment reconstitution

Splenocyte cell suspensions from noninfected *ifnγ* ^+/+^ *pfn* ^−/−^ or *ifnγ* ^−/−^ *pfn* ^+/+^ donor mice were enriched for CD8^+^ T-cells as described above and were used only when the CD8-enrichment was ≥98%. CD8^+^ T-cells (5×10^6^ cells / recipient mouse) were transferred intravenously into *cd8* ^−/−^ noninfected recipient mice. At two weeks after the adoptive cell transfer, the recipient mice were checked for CD8^+^ T-cell compartment reconstitution by sampling 50 µL of peripheral blood, which was analyzed by flow cytometry as described above. After the detection of CD8^+^ T-cells in the peripheral blood, the recipient mice were challenged with 100 bt from the Colombian strain of *T. cruzi*.

### Creatine-kinase detection

The activity of the creatine kinase cardiac MB isoenzyme (CK-MB), a myocardial injury marker, was measured using a commercial CK-MB Liquiform kit (Labtest, Brazil) in accordance with the manufacturer's recommendations. The incubation of sera samples with the substrate led to a net increase in the NADPH concentration that was directly proportional to the enzyme activity in the samples. The assay was adapted for reading in a microplate spectrophotometer (Microplate Reader Benchmark; Bio-Rad, Providence, RI, USA) to allow the study of small quantities of mouse serum in accordance with the manufacturer's recommendations. The optical density at 340 nm was recorded every 2 minutes for a duration of 15 minutes.

### Electrocardiogram (ECG) registers

All mice were intraperitoneally tranquilized with diazepam (20 mg/Kg) and the transducers were carefully placed subcutaneously according to chosen preferential derivation (DII). The traces were recorded during 2 minutes using a digital system Power Lab 2/20 that was connected to a bio-amplifier at 2 mV for 1 s (PanLab Instruments, Barcelona, Spain). Filters were standardized to between 0.1 and 100 Hz and traces were analyzed using the Scope software for Windows V3.6.10 (PanLab Instruments, Barcelona, Spain). We measured the heart rate (beats per minute, bpm), the duration of the P wave and QRS, ad PR and QT intervals in milliseconds (ms). The relationship between the QT interval and the RR interval in the mouse was assessed in all animals. To obtain physiologically relevant values for the heart rate-corrected QT interval (QTc) in units of time (rather than time to a power that is not equal to 1), the observed RR interval (RR0) was first expressed as a unitless multiple of 100 ms, yielding a normalized RR interval, RR100 = RR0/100 ms. Next, the value of the exponent (*y*) in the relationship QT0 = QTc×RR*y*100 was assessed, with QT0 indicating the observed QT (in ms) and the unit for QTc being milliseconds. The natural logarithm was computed for each side of this relationship [(QT0) = In (QTc)+*y*ln (RR100)]. Thus, the slope of the linear relationship between the log-transformed QT and RR100 defined the exponent to which the RR interval ratio should be raised to correct QT for heart rate [7].

### Statistical analysis

The data were expressed as the arithmetic mean ± SD. A student's *t* test was adopted to analyze the statistical significance of the apparent differences. The Kaplan-Meier method was employed to compare the survival rates of the groups. All statistical tests were performed using SPSS 8.0 software. Differences were considered statistically significant at *p*<0.05 (*).

## Supporting Information

Figure S1 **Lymphocyte populations infiltrating the cardiac tissue of** ***T. cruzi*** **-infected mice.** C57BL/6 mice were infected with 100 bt of the Colombian strain of *T. cruzi* and lymphocyte populations invading the cardiac tissue were evaluated by flow cytometry at 40 dpi. (**A**) Representative SSCxFSC profile of inflammatory cells isolated from cardiac tissue of *T. cruzi*-infected. R1 gated cells were analyzed for TCR and CD8 expression. (**B**) Histogram overlay of inflammatory cells (R1 gated) isolated from cardiac tissue of *T. cruzi*-infected labeled for TCR and isotype control. Dot plot of TCR^+^ cells (R2 gated) stained for CD4 and CD8. Dot plot of inflammatory cells (R1 gated) isolated from cardiac tissue of *T. cruzi*-infected labeled for NK1.1 and CD8. (**C**) Histogram of inflammatory cells (R1 gated) isolated from cardiac tissue of *T. cruzi*-infected labeled for CD8 (R2 gated) and TCR.
(TIF)

Figure S2 **Splenomegaly and increased number of spleen cells in** ***T. cruzi*** **-infected mice.** C57BL/6 mice were infected with 100 bt of the Colombian strain of *T. cruzi*. (**A**) Relative spleen weight (mg/g) and (**B**) splenocyte cellularity in noninfected (NI; pool of three age-matched controls per analyzed point) and *T. cruzi*-infected C57BL/6 mice at 15, 30, 45, 60, 90 and 120 dpi. Each circle represents an individual mouse. These data represent three independent experiments. ^*^, *p*<0.05; ^**^, *p*<0.01; and ^***^, *p*<0.001, comparing NI and *T. cruzi*-infected mice.
(TIF)

Figure S3 **IFNγ^+^IL-10^+^ cells prevailed in peripheral blood but not in cardiac tissue of** ***T. cruzi*** **-infected mice.** C57BL/6 mice were infected with 100 bt of the Colombian strain of *T. cruzi* and the presence of CD8^+^ expressing IFNγ and IL-10 in peripheral blood and cardiac tissue was evaluated by flow cytometry (**A**) Representative dot plots of flow cytometry analysis of peripheral blood CD8^+^ T-cells [R1 (SSCxFSC) /R2 (TCR)/R3 (CD8) gated] that were analyzed for IFNγ and IL-10 expression in *T. cruzi*-infected mice at 45 and 120 dpi. (**B**) Frequencies of double-stained CD8^+^IFNγ^+^IL-10^+^ and (**C**) CD8^+^IFNγ^+^IL-10^neg^ in peripheral blood T-cells of heart infiltrating cells (R1/R2 gated) in *T. cruzi*-infected mice at 45 and 120 dpi. (**D**) Representative histograms and dot plots of flow cytometry analysis of heart infiltrating CD8^+^ cells [R1 (SSCxFSC)/R2 (CD8) gated] expressing IFNγ and IL-10 in *T. cruzi*-infected mice at 45 dpi.
(TIF)

Figure S4 **Representative dot plots of CD8^+^IFNγ^+^ expressing CCR5 and LFA-1 in** ***T. cruzi*** **-infected mice.** C57BL/6 mice were infected with 100 bt of the Colombian strain of *T. cruzi* and the presence of CD8^+^IFNγ^+^ expressing CCR5 and LFA-1 in peripheral blood was evaluated by flow cytometry. Representative dot plots of flow cytometry analysis of peripheral blood cells (R1 gated) CD8^+^IFNγ^+^ (R6 gated) were analyzed for isotype controls or CCR5 and LFA-1in *T. cruzi*-infected mice at 45 dpi.
(TIF)

Figure S5 **CD8^+^ cells from** ***ifnγ*** **^−/−^** ***pfn*** **^+/+^ and** ***ifnγ*** **^+/+^** ***pfn*** **^−/−^** ***T. cruzi*** **-infected donors were activated.** C57BL/6, *ifnγ* ^−/−^ *pfn* ^+/+^ and *ifnγ* ^+/+^ *pfn* ^−/−^ mice were infected with 100 bt of the Colombian strain of *T. cruzi* and the phenotypic and functional characterization of the CD8^+^ used for cell transfer to C57BL/6 and *cd8* ^−/−^ infected recipients was evaluated by flow cytometry, at 20 dpi. (**A**) Increased spleen cellularity in *T. cruzi*-infected mice at 20 dpi. Representative histograms of flow cytometry analysis of splenocytes [R1 (SSCxFSC) /R2 (TCR) gated] that express TCR and CD8 in *T. cruzi*-infected mice at 20 dpi. (**B**) Representative dot plots of flow cytometry analysis of splenocytes [R1 (SSCxFSC)/R2 (CD8) gated] that were analyzed for expression of IFNγ, Pfn, IL-10, TNF and CD107a in *T. cruzi*-infected mice at 20 dpi. (**C**) Representative histograms of flow cytometry analysis of splenocytes (R1 gated) of CFSE^high^-based lymphoproliferative response after 72 hours of stimuli with anti-CD3 and anti-CD28 in noninfected and *T. cruzi*-infected mice at 20 dpi. The frequencies of cycling cells (R3 and R4 gated) are shown. (**D**) Representative dot plots of flow cytometry analysis of splenocytes (R1 gated) of CFSE^low^-based lymphoproliferative response after 72 hours of stimuli in *T. cruzi*-infected mice at 20 dpi. After stimulation, the cells were stained with APC-conjugated anti-CD8. The cycling CFSE^low^CD8^+^ cells were detected in gates R4 and R5. Representative flow cytometry profiles and means of three animals per analyzed group. This represents two independent experiments.
(TIF)

Figure S6 **Noninfected recipient** ***cd8*** **^−/−^ mice were reconstituted with CD8^+^ cells from noninfected donors.** Representative dot plots of flow cytometry analysis of peripheral blood cells (R1 gated) from *cd8* ^−/−^ mice non-reconstituted or reconstituted with CD8^+^ cells from naïve *ifnγ* ^+/+^ *pfn* ^−/−^ and *ifnγ* ^−/−^ *pfn* ^+/+^ donors at 15 days after cell reconstitution stained for CD4 and CD8 molecules.
(TIF)
