# Supplementary figures and images for: CD8+ T-Cells Expressing Interferon Gamma or Perforin Play Antagonistic Roles in Heart Injury in Experimental Trypanosoma Cruzi-Elicited Cardiomyopathy
Source: PLoS Pathog. 2012 Apr 19;8(4):e1002645. doi: 10.1371/journal.ppat.1002645 (PMC3330123; doi:10.1371/journal.ppat.1002645)

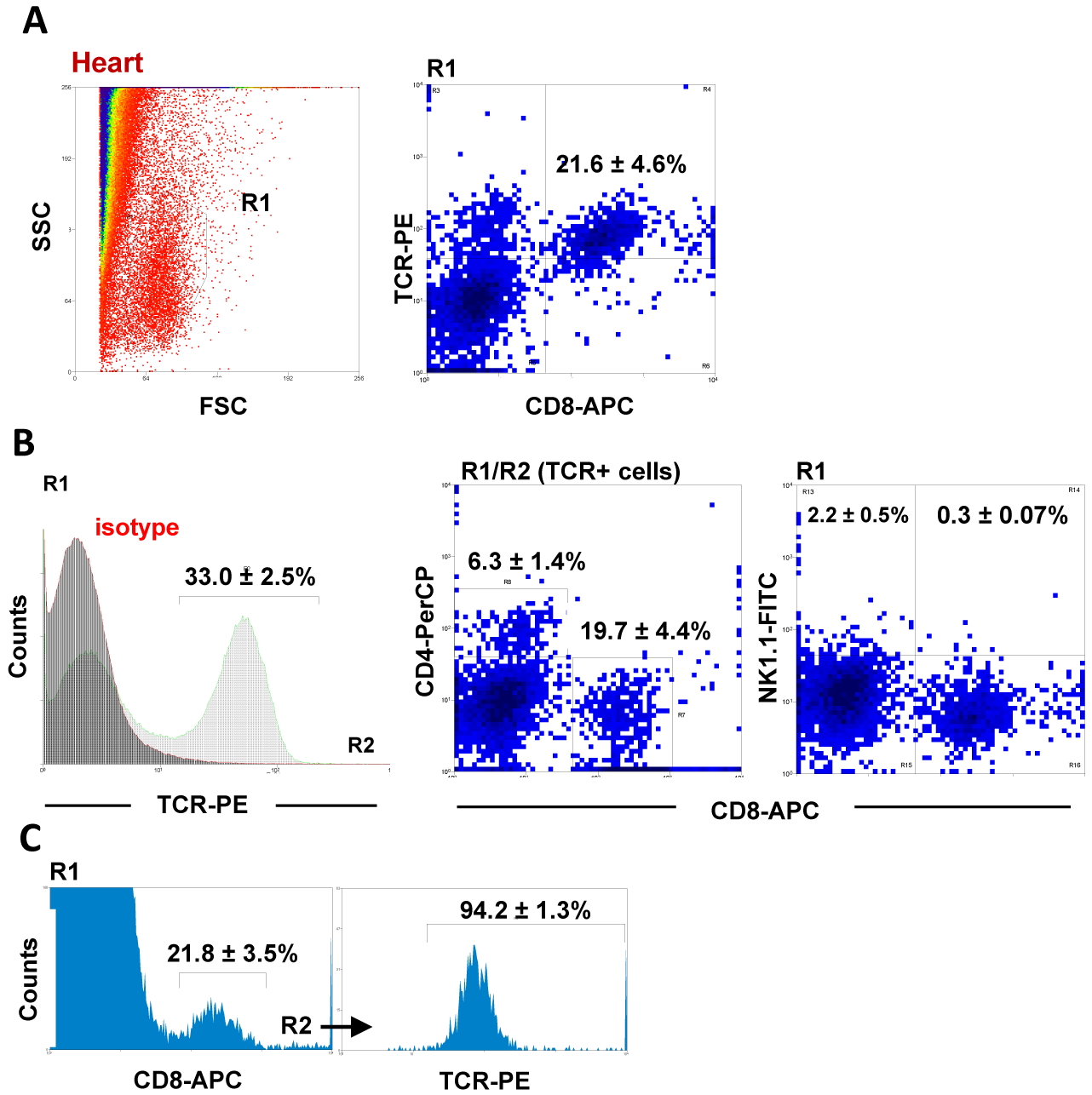

Supplement: Figure S1 — Lymphocyte populations infiltrating the cardiac tissue of T. cruzi -infected mice. C57BL/6 mice were infected with 100 bt of the Colombian strain of T. cruzi and lymphocyte populations invading the cardiac tissue were evaluated by flow cytometry at 40 dpi. (A) Representative SSCxFSC profile of inflammatory cells isolated from cardiac tissue of T. cruzi-infected. R1 gated cells were analyzed for TCR and CD8 expression. (B) Histogram overlay of inflammatory cells (R1 gated) isolated from cardiac tissue of T. cruzi-infected labeled for TCR and isotype control. Dot plot of TCR+ cells (R2 gated) stained for CD4 and CD8. Dot plot of inflammatory cells (R1 gated) isolated from cardiac tissue of T. cruzi-infected labeled for NK1.1 and CD8. (C) Histogram of inflammatory cells (R1 gated) isolated from cardiac tissue of T. cruzi-infected labeled for CD8 (R2 gated) and TCR. (TIF) [file ppat.1002645.s001.tif]

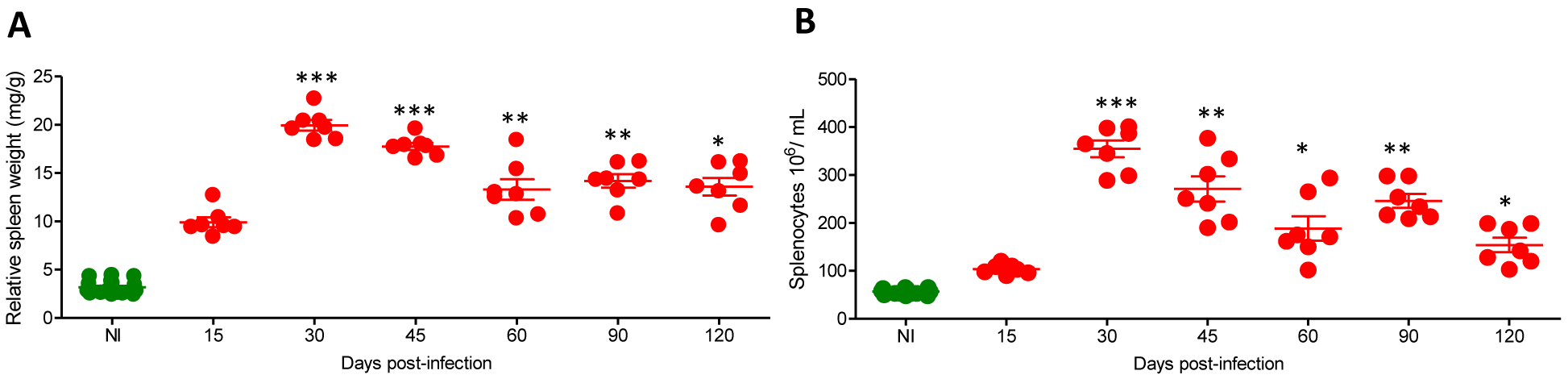

Supplement: Figure S2 — Splenomegaly and increased number of spleen cells in T. cruzi -infected mice. C57BL/6 mice were infected with 100 bt of the Colombian strain of T. cruzi. (A) Relative spleen weight (mg/g) and (B) splenocyte cellularity in noninfected (NI; pool of three age-matched controls per analyzed point) and T. cruzi-infected C57BL/6 mice at 15, 30, 45, 60, 90 and 120 dpi. Each circle represents an individual mouse. These data represent three independent experiments. *, p<0.05; **, p<0.01; and ***, p<0.001, comparing NI and T. cruzi-infected mice. (TIF) [file ppat.1002645.s002.tif]

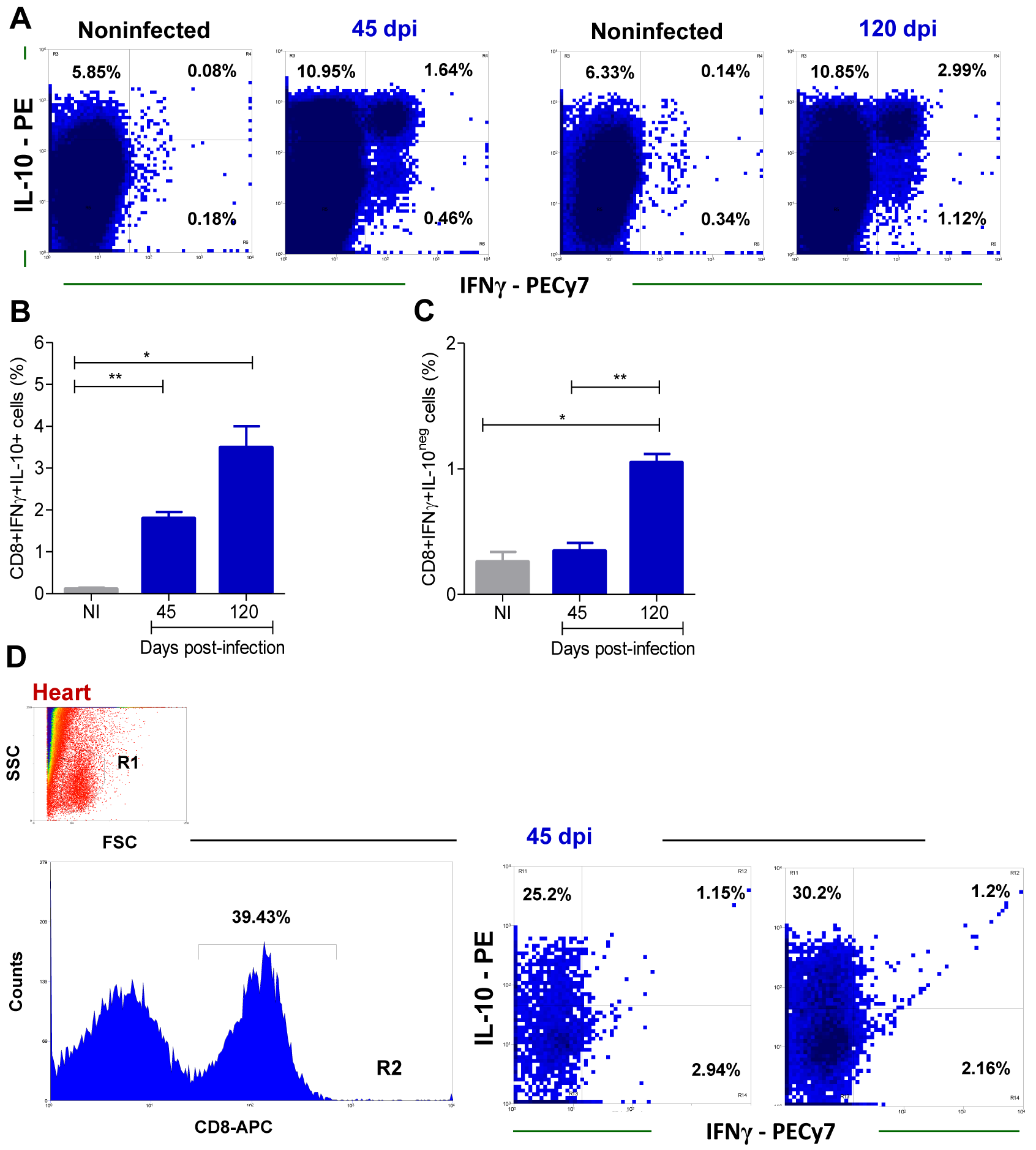

Supplement: Figure S3 — IFNγ+IL-10+ cells prevailed in peripheral blood but not in cardiac tissue of T. cruzi -infected mice. C57BL/6 mice were infected with 100 bt of the Colombian strain of T. cruzi and the presence of CD8+ expressing IFNγ and IL-10 in peripheral blood and cardiac tissue was evaluated by flow cytometry (A) Representative dot plots of flow cytometry analysis of peripheral blood CD8+ T-cells [R1 (SSCxFSC) /R2 (TCR)/R3 (CD8) gated] that were analyzed for IFNγ and IL-10 expression in T. cruzi-infected mice at 45 and 120 dpi. (B) Frequencies of double-stained CD8+IFNγ+IL-10+ and (C) CD8+IFNγ+IL-10neg in peripheral blood T-cells of heart infiltrating cells (R1/R2 gated) in T. cruzi-infected mice at 45 and 120 dpi. (D) Representative histograms and dot plots of flow cytometry analysis of heart infiltrating CD8+ cells [R1 (SSCxFSC)/R2 (CD8) gated] expressing IFNγ and IL-10 in T. cruzi-infected mice at 45 dpi. (TIF) [file ppat.1002645.s003.tif]

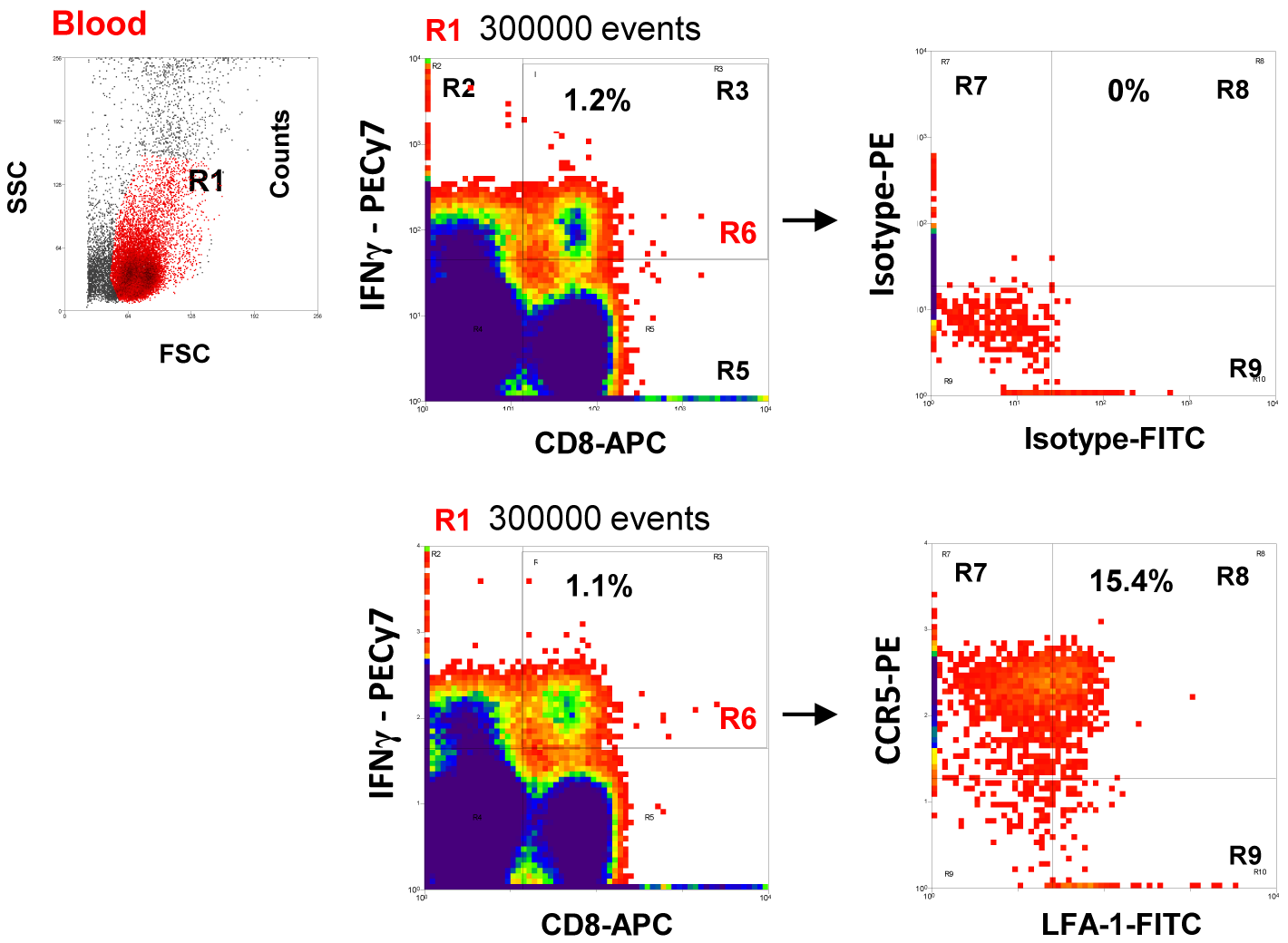

Supplement: Figure S4 — Representative dot plots of CD8+IFNγ+ expressing CCR5 and LFA-1 in T. cruzi -infected mice. C57BL/6 mice were infected with 100 bt of the Colombian strain of T. cruzi and the presence of CD8+IFNγ+ expressing CCR5 and LFA-1 in peripheral blood was evaluated by flow cytometry. Representative dot plots of flow cytometry analysis of peripheral blood cells (R1 gated) CD8+IFNγ+ (R6 gated) were analyzed for isotype controls or CCR5 and LFA-1in T. cruzi-infected mice at 45 dpi. (TIF) [file ppat.1002645.s004.tif]

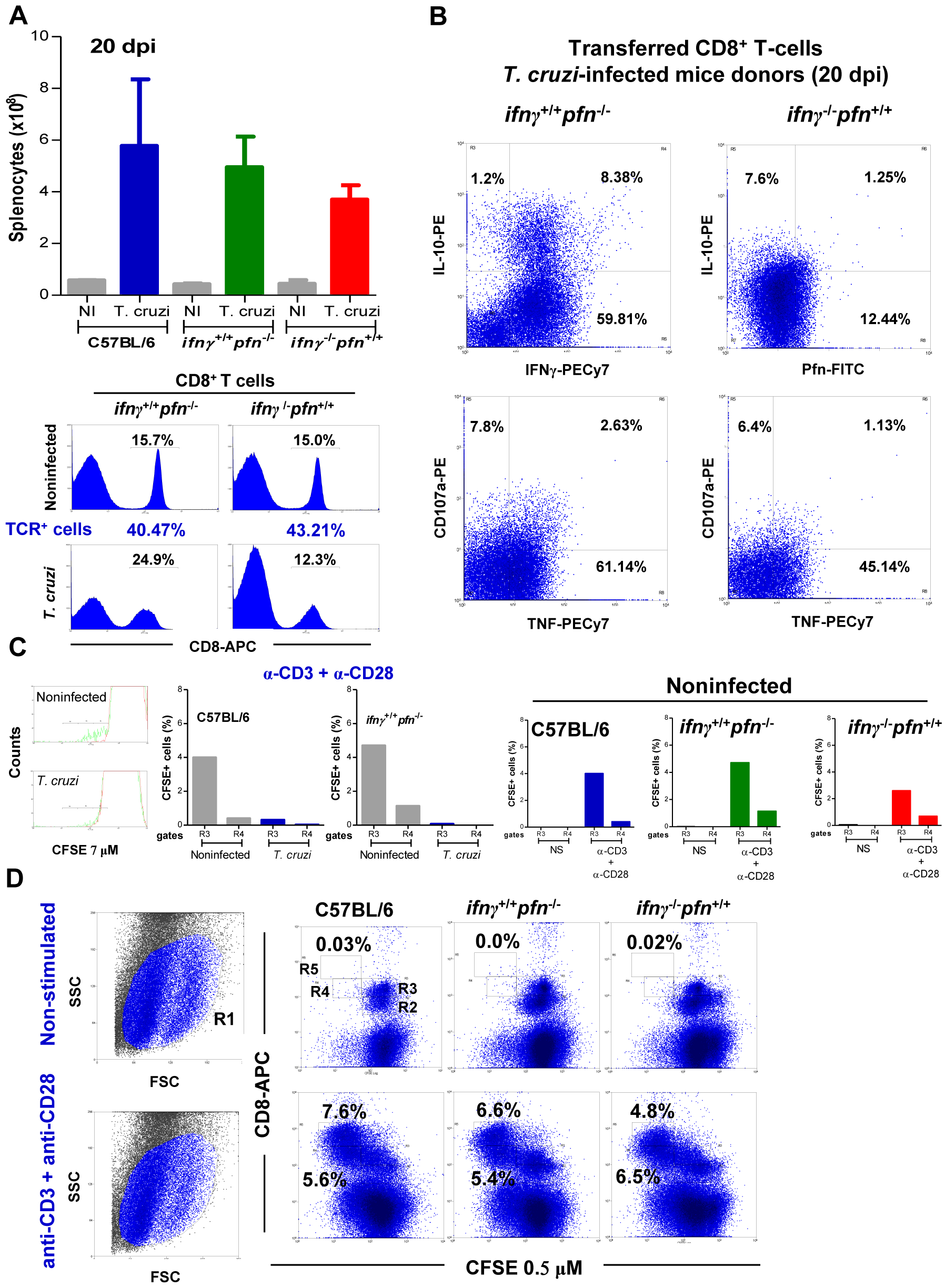

Supplement: Figure S5 — CD8+ cells from ifnγ −/− pfn +/+ and ifnγ +/+ pfn −/− T. cruzi -infected donors were activated. C57BL/6, ifnγ −/− pfn +/+ and ifnγ +/+ pfn −/− mice were infected with 100 bt of the Colombian strain of T. cruzi and the phenotypic and functional characterization of the CD8+ used for cell transfer to C57BL/6 and cd8 −/− infected recipients was evaluated by flow cytometry, at 20 dpi. (A) Increased spleen cellularity in T. cruzi-infected mice at 20 dpi. Representative histograms of flow cytometry analysis of splenocytes [R1 (SSCxFSC) /R2 (TCR) gated] that express TCR and CD8 in T. cruzi-infected mice at 20 dpi. (B) Representative dot plots of flow cytometry analysis of splenocytes [R1 (SSCxFSC)/R2 (CD8) gated] that were analyzed for expression of IFNγ, Pfn, IL-10, TNF and CD107a in T. cruzi-infected mice at 20 dpi. (C) Representative histograms of flow cytometry analysis of splenocytes (R1 gated) of CFSEhigh-based lymphoproliferative response after 72 hours of stimuli with anti-CD3 and anti-CD28 in noninfected and T. cruzi-infected mice at 20 dpi. The frequencies of cycling cells (R3 and R4 gated) are shown. (D) Representative dot plots of flow cytometry analysis of splenocytes (R1 gated) of CFSElow-based lymphoproliferative response after 72 hours of stimuli in T. cruzi-infected mice at 20 dpi. After stimulation, the cells were stained with APC-conjugated anti-CD8. The cycling CFSElowCD8+ cells were detected in gates R4 and R5. Representative flow cytometry profiles and means of three animals per analyzed group. This represents two independent experiments. (TIF) [file ppat.1002645.s005.tif]

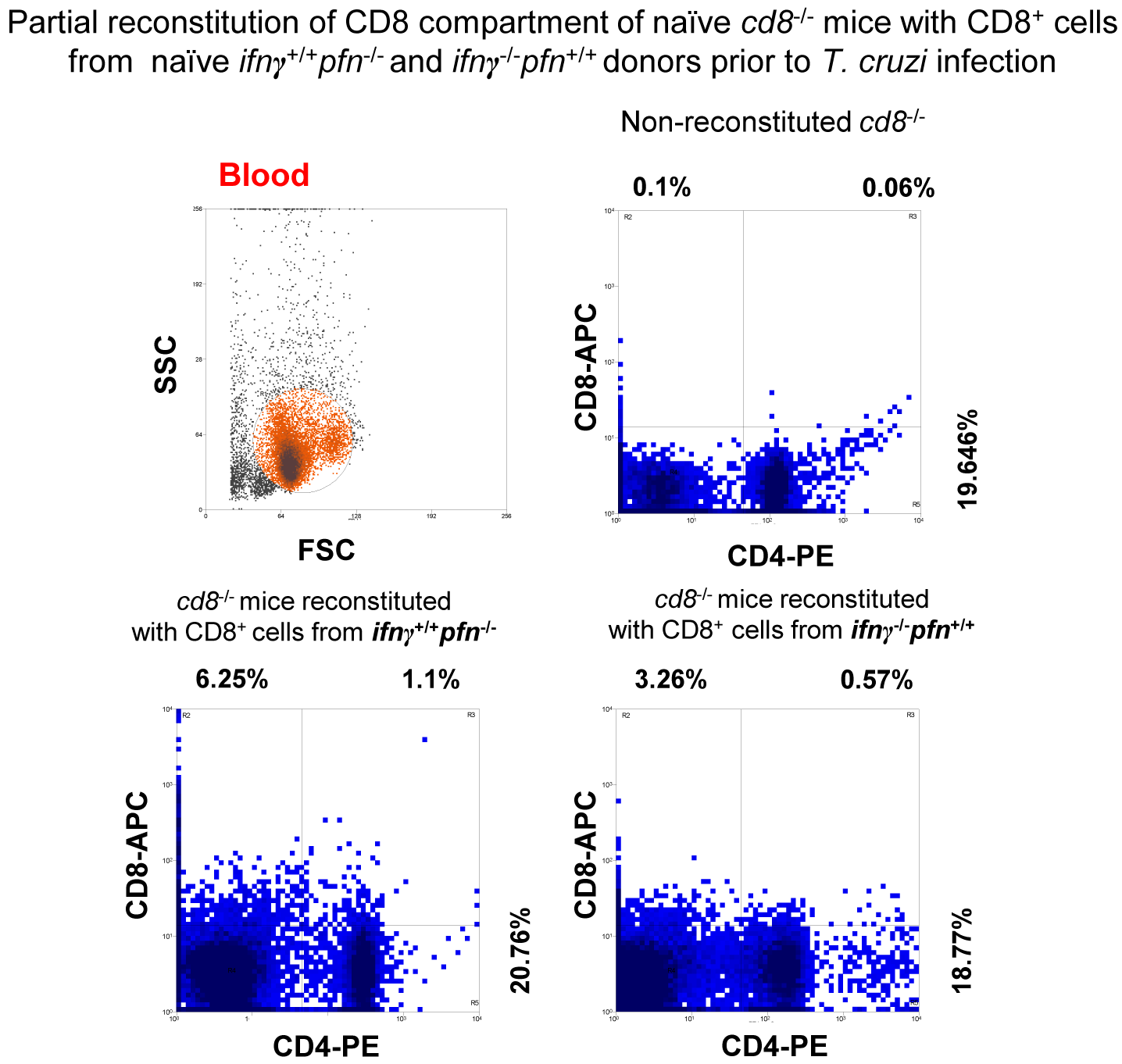

Supplement: Figure S6 — Noninfected recipient cd8 −/− mice were reconstituted with CD8+ cells from noninfected donors. Representative dot plots of flow cytometry analysis of peripheral blood cells (R1 gated) from cd8 −/− mice non-reconstituted or reconstituted with CD8+ cells from naïve ifnγ +/+ pfn −/− and ifnγ −/− pfn +/+ donors at 15 days after cell reconstitution stained for CD4 and CD8 molecules. (TIF) [file ppat.1002645.s006.tif]
